# Linguistic inputs must be syntactically parsable to fully engage the language network

**DOI:** 10.1101/2024.06.21.599332

**Published:** 2024-06-21

**Authors:** Carina Kauf, Hee So Kim, Elizabeth J. Lee, Niharika Jhingan, Jingyuan Selena She, Maya Taliaferro, Edward Gibson, Evelina Fedorenko

**Affiliations:** 1 Department of Brain and Cognitive Sciences, Massachusetts Institute of Technology, Cambridge, MA 02139 USA; 2 McGovern Institute for Brain Research, Massachusetts Institute of Technology, Cambridge, MA 02139 USA; 3 Department of Psychology, New York University, New York, NY 10012 USA; 4 The Program in Speech and Hearing Bioscience and Technology, Harvard University, Cambridge, MA 02138 USA

## Abstract

Human language comprehension is remarkably robust to ill-formed inputs (e.g., word transpositions). This robustness has led some to argue that syntactic parsing is largely an illusion, and that incremental comprehension is more heuristic, shallow, and semantics-based than is often assumed. However, the available data are also consistent with the possibility that humans always perform rule-like symbolic parsing and simply deploy error correction mechanisms to reconstruct ill-formed inputs when needed. We put these hypotheses to a new stringent test by examining brain responses to a) stimuli that should pose a challenge for syntactic reconstruction but allow for complex meanings to be built within local contexts through associative/shallow processing (sentences presented in a backward word order), and b) grammatically well-formed but semantically implausible sentences that should impede semantics-based heuristic processing. Using a novel behavioral syntactic reconstruction paradigm, we demonstrate that backward-presented sentences indeed impede the recovery of grammatical structure during incremental comprehension. Critically, these backward-presented stimuli elicit a relatively low response in the language areas, as measured with fMRI. In contrast, semantically implausible but grammatically well-formed sentences elicit a response in the language areas similar in magnitude to naturalistic (plausible) sentences. In other words, the ability to build syntactic structures during incremental language processing is both necessary and sufficient to fully engage the language network. Taken together, these results provide strongest to date support for a generalized reliance of human language comprehension on syntactic parsing.

## 1. Introduction

Every day, humans produce and understand sentences they have never encountered before. This expressive power of language results from its compositionality: sentence meanings depend on the meanings of their constituent words and the ways in which the words relate to one another in the sentence’s structure. In particular, because sentence structure can be systematically decoded from the word order and/or morphosyntactic markers, we can understand novel inputs, even implausible/nonsensical ones, like the famous ‘*Colorless green ideas sleep furiously*’ example (Chomsky, 1957). However, the computations that enable us to quickly assemble complex meanings as we process language remain debated.

According to one view, humans perform ***rule-like symbolic parsing*** on linguistic inputs to derive complex meanings. Support for this view comes from strong sensitivity of human language processing mechanisms to structure. For example, a telltale signature of the language brain areas (Fedorenko et al., 2024) is a stronger response to structured stimuli, like sentences, compared to unstructured word-lists (Fedorenko et al., 2010; Pallier et al., 2011; Diachek et al., 2020; Shain, Kean et al., 2024), presumably because sentences engage computations related to structure building. This sensitivity to structure extends to even meaningless stimuli—so-called Jabberwocky sentences, like ‘*Twas brillig and the slithy toves did gyre and gimble in the wabe…*’ (Carroll, 1872)—compared to unstructured nonword-lists, although the overall response to nonword-composed stimuli is lower (Humphries et al., 2006; Fedorenko et al., 2010, 2016; Goucha & Friederici, 2015; Matchin et al., 2017; Shain, Kean et al., 2024). Another line of evidence comes from stronger responses in the language areas to more syntactically complex structures, including complexity associated with i) integrating words into a memory representation of the context (Holcomb, 1993; Ben-Shachar et al., 2003; Constable et al., 2004; Lau et al., 2006; Fedorenko et al., 2013; Blank et al., 2016; Shain et al., 2022; behavioral evidence: Gibson, 2000; Lohse et al., 2004; Grodner & Gibson, 2005) and ii) processing structurally unexpected elements (Lopopolo et al., 2017; Fedorenko et al., 2020; Shain, Blank et al., 2020; Heilbron et al., 2022; Goldstein et al., 2022; Wang et al., 2023; behavioral evidence: Demberg & Keller, 2008; Levy, 2008b; Smith & Levy, 2013; Shain et al., 2024).

An alternative family of views hold that human language comprehension may be more heuristic, shallow, and semantics-based than is often assumed (Ferreira et al., 2002; Sanford & Sturt, 2002; Tabor & Hutchins, 2004; Kim & Osterhout, 2005; Swets et al., 2008; Frank & Bod, 2011; Kuperberg, 2016; Mahowald et al., 2023). For example, Mollica, Siegelman et al. (2020) used fMRI to measure the language areas’ response to sentences with local word-order swaps (e.g., ‘*messages and gifts’→‘and messages gifts’*) and found that such manipulations do not decrease the response relative to well-formed sentences, even when sentences become quite syntactically degraded. Only when word swaps involved far-away words and destroyed local semantic dependencies, did the language areas’ response decrease. The authors therefore argue that interpretation can proceed without syntactic analysis, because our past linguistic experience tells us which words typically combine *semantically*. Thus, the fundamental computation of the language brain areas is ***syntax-independent semantic composition***.

However, Mollica, Siegelman et al.’s (2020) findings, and other evidence for syntax-independent semantic composition, have another explanation: humans may show robustness (and correspondingly strong responses in the language areas) to ill-formed inputs because they readily deploy error correction mechanisms (Gibson et al., 2013; Levy, 2008a, 2011; Levy et al., 2009; Zhang et al., 2024). Under this view, the human language system still performs syntax-driven composition, but syntactically corrupted input first needs to be reconstructed. Indeed, psycholinguistic studies have provided ample support for the human ability to cope with diverse errors, including word-order errors (Erickson & Mattson, 1981; Ferreira & Stacey, 2000; Ferreira & Bailey, 2004; Levy, 2008a; Mirault et al., 2018; Ryskin et al., 2018; Wen et al., 2021).

Here we test the necessity and sufficiency of syntactic processing for driving the brain’s response to language and find that the language network is fully engaged whenever syntactic structures can be built during incremental processing. These results argue against the shallower construals of linguistic computations.

## 2. Methods

**Figure 1.**
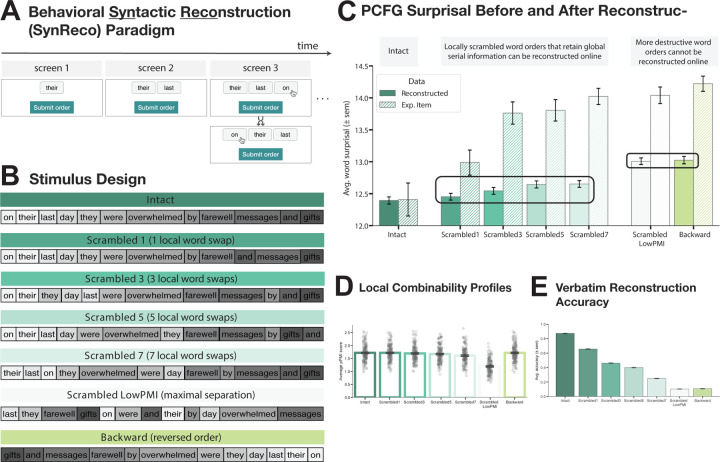
Word-order scrambled stimuli that elicit a sentence-level response in the language network are amenable to real-time syntactic reconstruction. **A)** Illustration of the novel behavioral Syntactic Reconstruction (SynReco) paradigm. Sentences are revealed on the screen one word at a time, in boxes with a drag-and-drop function. At each time step, participants can reorder the words on the screen into their best guess of the most likely word order. Each newly revealed word is appended to the participant’s last submitted order. **B)** A sample item from the critical experiment (figure adapted from Mollica, Siegelman et al., 2020); the grayscale color gradient is used to illustrate the increasing degradedness (i.e., the color spectrum becomes progressively more discontinuous with more swaps, but is preserved in the Backward condition). **C)** Average grammaticality of (i) the experimental materials (hatched bars) and (ii) participant reconstructions (solid bars) across conditions. Grammaticality is quantified as average PCFG word surprisal. Locally scrambled conditions are amenable to real-time reconstruction (PCFG surprisal following reconstruction is comparable to the *Intact* condition), but more severely degraded conditions (Scrambled *LowPMI* and *Backward*) are difficult to reconstruct in real time. **D)** Average positive PMI scores for experimental materials (see Figure SI 4 for directional PPMI results). **E)** Average rate of recovery of the verbatim unscrambled input (reconstruction accuracy) after incremental parsing with the novel behavioral paradigm (verbatim reconstruction patterns replicate the results reported in Mollica, Siegelman et al., but as the fMRI results show, the PCFG surprisal estimates of the reconstructed strings (Figure 1C, solid bars), not the verbatim reconstruction accuracies, mirror the responses in the language network).

Our approach is two-fold. First, we develop a novel behavioral paradigm that allows us to assess the ability to repair and interpret syntactically ill-formed input during incremental, word-by-word processing. Many past studies have examined the *costs* associated with the presence of syntactic violations using behavioral and neural measures (e.g., Osterhout & Holcomb, 1992; Hagoort et al., 1993; Newman et al., 2001; Kuperberg et al., 2003; De Vincenzi et al., 2003; Ditman et al., 2007; Friederici et al., 2010; Nieuwland et al., 2013). Other studies have investigated the *offline interpretation* of ill-formed linguistic inputs, where participants are presented with such inputs and have to answer questions that probe their interpretation of the sentence meaning or are asked to re-arrange the words into their most likely order (e.g., Ferreira & Stacey, 2000; Gibson et al., 2013; Chen et al., 2023, Mollica, Siegelman et al., 2020). However, to our knowledge, no method currently exists for evaluating how participants may be *interpreting ill-formed sequences as they process them incrementally*. We developed an approach where participants receive stimuli word by word (visually, on a computer screen) and, after each new word is uncovered, have the ability to reorder the words that are currently on display if they think the order in which they appear does not make sense (Figure 1A). By examining the orders that participants consider at different points in the sequence, we can make inferences about the syntactic structures they are building and the interpretations they are deriving. Using this new paradigm, we show that the stimuli with local word-order scrambling from Mollica, Siegelman et al.’s (2020) study, which elicit a strong response in the language brain areas, are amenable to real-time syntactic reconstruction, in contrast to conditions with more severe word-order rearrangement.

And second, using fMRI, we examine responses in the language areas to several linguistic conditions, including two critical conditions: (i) sentences presented backwards, from the last word to the first, and (ii) nonsensical but grammatically well-formed sentences. Sentences presented backwards allow for local semantic composition but should make syntactic reconstruction in real time challenging. In particular, reversing the words in a sentence does not disrupt local inter-word dependencies, so a processor that does not depend on word order should be able to perform local semantic composition. Consequently, the syntax-independent semantic composition hypothesis predicts that the response in the language areas should be as high as to grammatical sentences. However, the consistent reversal of the order of constituent elements intuitively places a substantial burden on the parsing mechanisms, at least in a language that relies heavily on word-order cues (as confirmed empirically, using our novel behavioral paradigm described above). Therefore, the syntax-dependent composition hypothesis predicts a low response in the language areas.

In contrast, nonsensical but grammatically well-formed sentences (like the ‘*Colorless green ideas…*’ example; Chomsky, 1957) allow for syntactic structure building, but building complex meanings is impeded by semantic implausibility. As noted in the Introduction, the response in the language areas to typical (well-formed and plausible) sentences is higher than to Jabberwocky sentences, which are syntactically well-formed but made up of nonwords (Humphries et al., 2005; Fedorenko et al., 2010; Shain, Kean et al., 2024). But is the stronger response to typical sentences due to i) the presence of real words, which are the stored basic units of language and contain critical cues for structure building (e.g., Pollard & Sag, 1994; Steedman, 2000; Goucha & Friederici, 2015), or ii) the plausibility of the resulting meanings, which can aid top-down semantic prediction (e.g., Kutas & Hillyard, 1984; Federmeier & Kutas, 1999; Nieuwland & Van Berkum, 2006; Bicknell et al., 2010; Matsuki et al., 2011)? The syntax-independent semantic composition hypothesis, whereby interpretation proceeds ‘bottom up’ from local semantic associations, predicts a low response to nonsensical sentences in the language areas because our **language processor should not attempt to form complex meanings from words that are unlikely to enter into a semantic dependency** (based on our prior linguistic experience and world knowledge). In contrast, the syntax-dependent semantic composition hypothesis predicts a strong response given that semantic composition can still take place even if the resulting meaning is implausible. If it turns out that the language areas’ computations are not affected by plausibility, this would open up new questions about where in the brain plausibility is computed.

### 2.1 Experiment 1: Behavioral incremental syntax reconstruction study

In this experiment, we assessed people’s ability to reconstruct syntactically ill-formed input during incremental word-by-word processing.

#### 2.1.1 Design and materials

We developed a novel behavioral paradigm (SynReco for “syntax reconstruction”) in which sentences are revealed one word at a time, with each word presented within a box that has a drag-and-drop function. At each time step, participants are asked to reorder the words on the screen according to their best guess as to the most likely word order, even when the current displayed sentence fragment does not yet form a grammatical sequence. Participants can reorder as many words as they want, until they are satisfied with the resulting order. Once they are satisfied with the order, they can click a “Submit order” button to reveal the next word or, if the last word of the stimulus has already been revealed, to end the trial.

The experiment included intact sentences and different versions of altered-word-order sentences. The critical stimuli consisted of 35 items, each in 7 versions (corresponding to conditions), for a total of 245 stimuli. The stimuli were adopted from Mollica, Siegelman et al. (2020). As described in greater detail in Mollica, Siegelman et al. (2020), a set of 12-word-long sentences (the **Intact** condition) were extracted from the British National Corpus (BNC; Leech, 1992), of which we used a subset of 35 sentences. For consistency with the materials used in our fMRI study (see Section 2.2.1), we converted the word spellings to American English (e.g., *moustache → mustache*). Five of the six altered-word-order conditions came directly from Mollica, Siegelman et al. (2020): The locally scrambled conditions, **Scrambled{1,3,5,7}**, were created by iteratively and randomly choosing 1, 3, 5, or 7 words in each original sentence and swapping them with one of their immediate word neighbors. As reported in Mollica, Siegelman et al. (2020), these local word-swap manipulations, even for the 7-swap case, typically preserve local semantic dependency structure, as can be measured by pointwise mutual information (PMI) among nearby words (as detailed in Section 2.2.1). The **Scrambled_LowPMI** condition minimizes the combinability among nearby words and was created by placing content words that are adjacent/proximal in the original sentence far away from each other. In addition to these six conditions, we included a novel (**Backward**) condition, which was created by reversing the word order in the original sentences. *Backward* sentences are characterized by the same local semantic dependency structure as their *Intact* counterparts (Figure 1D, Figure SI 1, Table SI 1). The 245 stimuli were distributed across 7 experimental lists (35 stimuli each, 5 per condition) such that each list contained only one condition of an item. Each participant completed only one list.

Each list additionally included 7 practice items, and 19 filler items (7 of which were designed to be easier than the critical stimuli and served as attention checks). These items differed from the critical items in length (mean = 7 words, SD = 1.5), but, similar to the critical items, they were either grammatically well-formed or contained one or several local word swaps. We varied the number of words in these non-critical items in order to prevent participants from relying on their expectation of how many words are still to come in a sequence while performing the critical word-reordering task.

#### 2.1.2 Procedure

The experiment was implemented as a new JQuery module within the Ibex web-based psycholinguistic experiment software platform (https://github.com/addrummond/ibex).

The experiment began with detailed instructions. To encourage the careful reading of these instructions, we divided the information across three screens and allowed participants to advance to the next screen only after a 15-second delay, when a “Continue” button appeared. Participants were told that their task is to try to create a grammatical word order at each point during the trial. They were encouraged to try their best before moving on to reveal the next word, but they were also told that if—at some point in the trial—they cannot find a way to reorder the words so as to form a grammatical string, they should reveal the next word to see if that helps. For example, if the first two words are “this a”, reordering them does not help (“a this” is not a grammatical string), but the next word may be “is”, in which case a grammatical string can now be formed (“this is a”). Participants were informed that the words in each trial could always be reordered into (at least one) fully grammatical sentence (i.e., the original version of the sentence) and were advised that (i) on some trials no reordering may be needed, and that (ii) some trials might prove challenging. They were also told that in some cases, there may be multiple ways to order the words so as to create a grammatical string (e.g., “books and pencils” vs. “pencils and books”), and that in such cases, any of the permissible word orders would be accepted. Finally, they were warned that the experiment includes several (unmarked) attention check items—used to assess and maintain participants’ attentiveness throughout the experiment—and that consistent failure on these items might lead to exclusion from the experiment, whereas consistently good performance throughout the experiment would lead to a small bonus payment.

Following the instructions, participants completed 7 practice items. All stimuli were presented in lowercase letters and without punctuation. For the practice items, i) participants received feedback at each time step telling them if the submitted word order was permissible or not, and ii) the next word in the sequence was only revealed once participants had submitted one of the permissible word orders for the current time step. To be able to do this, we had to determine all grammatically allowed word orders at each time step for each of these items (as well as for the filler items—see below). C.K. and J.S. made an initial set of judgments about the word orders, and these judgements were then validated in an independent experiment (n = 23 participants). In particular, participants completed the experiment in the same experimental procedure as the critical experiment, and J.S. manually reviewed the set of unique orders submitted by the participants at each time step to include any additional grammatically allowed word orders that were missing from the initial set of permissible orders. In this way, the practice items served to train participants to submit their guess of the most likely word order *at each time step*, even if that guess may turn out to be wrong later on, instead of revealing multiple words at once before starting to reorder them.

Upon the completion of the practice items, the critical experiment began. For the critical items, participants did not receive any feedback; for the filler items, which were randomly interspersed with the critical items, they received feedback. In particular, for the subset of the filler items that served as attention checks, participants were notified when they submitted an ungrammatical word order at any point in the trial, to encourage them to carefully arrange the words at each time step. If they submitted grammatical orders at each time step, they were notified at the end of the trial that they passed an attention check. For the remaining filler items, participants were informed of their performance (pass or fail) when they submitted the final word order. The average completion time for this experiment was ~50 min.

#### 2.1.3 Participants

We recruited 140 participants through the Prolific web-based testing platform, restricting our task to participants with IP addresses in the United States. Participants were included in the analyses if they satisfied all of the following criteria: (i) they succeeded on at least 4 of the 7 attention check items (see above for details), and (ii) they succeeded on at least 6 of the 12 remaining filler items (see above for details). The numbers for i and ii were determined based on the error distributions (excluding the lowest quartile of participants, see Figure SI 2). Data from 72 participants were included in the final analysis.

### 2.2 Experiment 2: fMRI study

Each participant completed (i) the critical task, (ii) a language network localizer task, which was used to identify language-responsive brain regions (Fedorenko et al., 2010), and (iii) a localizer task for another network: the domain-general Multiple Demand (MD) network (Duncan, 2010), which was used in some control analyses. Most participants also completed one of two tasks for unrelated studies. The scanning sessions lasted approximately two hours.

#### 2.2.1 Critical task design and materials

Participants passively read 12-word-long stimuli in a blocked design. The stimuli were presented one word/nonword at a time and belonged to one of eight conditions: (1) Intact plausible sentences (S), (2) Backward sentences (BS), (3) Nonsense sentences (NS), (4) Jabberwocky sentences (JS), (5) Word lists (WL), (6) Nonword lists (NWL), and two conditions used to address a distinct research question ((7–8) Predictable and unpredictable phrase lists; see Discussion).

The stimuli for the sentence conditions consisted of 192 items, each in 4 versions (corresponding to conditions: S, BS, NS, and JS), for a total of 768 stimuli. The base set (for the **Intact plausible sentences (S)** condition) consisted of 140 sentences adopted from Mollica, Siegelman et al. (2020) and an additional set of 52 sentences. As described in greater detail in Mollica, Siegelman et al. (2020), a set of 12-word-long sentences were extracted from the British National Corpus (BNC; Leech, 1992), of which we used a subset of 140 sentences (the remaining 10 sentences in the Mollica, Siegelman et al.’s study contained a high proportion of function words, which presented a challenge in creating satisfactory *Nonsense* sentence variants; see below for details). We additionally extracted a set of 52 12-word-long sentences from the same corpus. We made minor adjustments to some of the sentences by converting the word spellings to American English (e.g., *moustache → mustache*).

To create the **Backward sentences (BS)** condition, we reversed the order of the words in each of the 192 sentences. A critical design feature of the stimuli in this condition is that they are characterized by the same local syntactic and semantic combinability of nearby words as the original sentences (see Figure 1D; Figure SI 2).

To create the **Nonsense sentences (NS)** condition, we first created a set of candidate nonsense sentences, where for each of the 192 sentences, we replaced each content word (noun, verb, adjective, and adverb) with a replacement word. Suitable replacement words were determined based on morpho-syntactic feature overlap with the word to be replaced. Specifically, we classified each word in each sentence according to its part-of-speech tag, syntactic dependency label, and morphological features (such as case, number, mood, or tense information), all obtained using the NLTK (Loper & Bird, 2002) and spaCy (Honnibal et al., 2020) models. Verbs were further subcategorized according to their argument valence, to approximate the linguistic contexts in which they could serve as possible replacements for other verbs. Nouns were additionally annotated with coarse phonological features, such as whether they began with a vowel or a consonant sound (determined via their orthography as a proxy) to ensure that the replacement nouns would have the right onset when following indefinite determiners. Based on these annotations, we created a “dictionary” that mapped a given feature set to a list of all content word tokens (with duplicates; e.g., if a word “cat” occurred twice in the original set of sentences, it was included twice in the dictionary) and consisted of 1,398 content words. We then iterated through the sentences and replaced each content word with a randomly sampled word from our dictionary that corresponded to the original word in terms of its annotated features. In this way, the component words were the same between the S and the NS conditions. The algorithm was largely successful based on examining the resulting sentences; however, we carefully hand-checked the algorithmically-created nonsense sentences to ensure that the category selectivity of the verbs was satisfied, because the unique distributional signature of each verb could only be coarsely approximated by the feature annotations. Hence, if needed, we chose another replacement word from the dictionary.

To create the **Jabberwocky sentences (JS)** condition, for each of the 192 sentences, we replaced each content word with a suitable replacement nonword. Suitable replacement nonwords were generated using the *generate classic* method for English from the Wuggy pseudoword generator python package (Keuleers & Brysbaert, 2010). This algorithm creates nonwords by matching the original word in terms of sub-syllabic structure and syllable-transition frequencies. The latter constraint ensures the preservation of functional morphology (e.g., the past tense marker -ed or the plural -s), because replacing these high-frequency syllables typically involves a massive change in transition frequency (Keuleers & Brysbaert, 2010). We then iterated through the sentences and replaced each content word with a matching nonword.

To create the stimuli for the **Word lists (WL)** condition, we gathered all words across the 192 sentences and randomly recombined these 2,304 words (192 sentences, each 12 words long) into 192 sequences of 12 words each (via sampling without replacement). To create the stimuli for the **Nonword lists (NWL)** condition, we gathered all words and nonwords across the 192 Jabberwocky sentences and randomly recombined these 2,304 words/nonwords into 192 sequences of 12 words/nonwords each (via sampling without replacement). The stimuli for the remaining two conditions, which were used to address a distinct question (see Discussion), consisted of 12-word-long sequences, each made up of six determiner-noun phrases of the form ‘the noun’ (see Figure SI 13 for details).

The stimuli for the sentence conditions (S, BS, NS, JS; 192 stimuli per condition)—where correspondence exists among the different-condition versions of the same sentence—were distributed across four experimental lists, such that each list contained only one version (S, BS, NS, or JS) of a given sentence and 48 stimuli for each of the four sentence conditions. In addition to the 192 sentence stimuli, each list included 48 Word Lists (WL), 48 Nonword Lists (NWL), and 96 items across two conditions that are not relevant to the current study (see Discussion), for a total of 384 stimuli. Each participant completed only one list.

**Figure 2.**
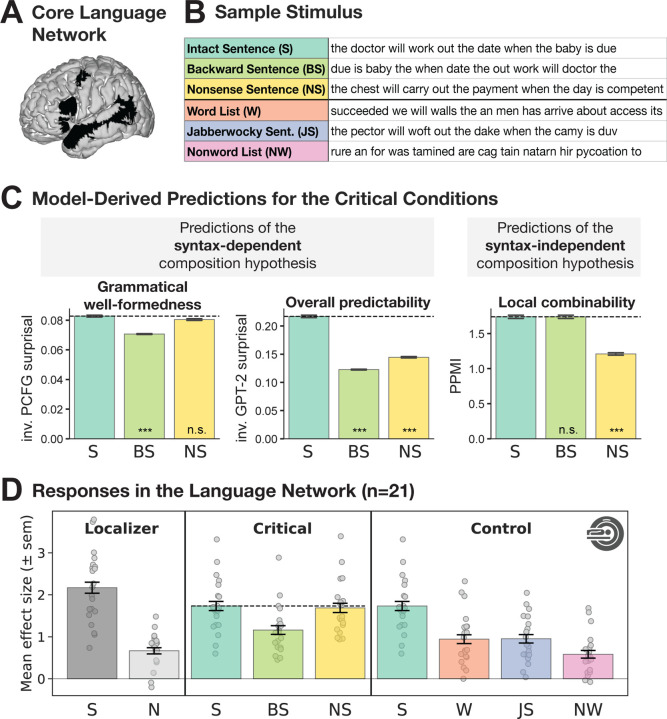
Syntax-driven meaning composition drives the language network’s response. **A)** The parcels that were used to functionally define the language-responsive areas in individual participants (Fedorenko et al., 2010). In each participant, the top 10% of most localizer-responsive (S>N) voxels within each parcel were taken as that participant’s region of interest. **B)** A sample item from the critical experiment. **C)** Quantitatively derived predictions for syntax-dependent vs. syntax-independent semantic composition hypotheses. The syntax-dependent panel is split up into predictions derived via structure-mediated vs. expectation-mediated incremental processing models (see Discussion). To match the expected direction of the neural responses in the language network, we show inverse surprisal (i.e., the reciprocal of surprisal) for the PCFG and GPT-2 models. Significant difference to the *Sentence* condition was established via post hoc pairwise t-tests, with p-values corrected for multiple comparisons using the Bonferroni procedure. **D)** Neural responses (in % BOLD signal change relative to fixation) to the conditions of the language localizer and critical and control experimental conditions within the language network (averaged across the five regions; the profiles of individual fROIs are similar, as shown in Figure 3). Dots show individual subject responses; error bars show standard errors of the mean by participants. The observed response pattern is best explained by syntax-*dependent* semantic composition (see panel C).

Prior to the experiment, we ensured that stimuli across conditions did not differ in low-level features, such as word frequencies or word lengths (see Figure SI 3). We additionally evaluated the stimuli to ensure that they have the desired properties for dissociating the syntax-dependent and the syntax-independent semantic composition hypotheses, with a focus on the two critical conditions (see Figure 2C): *Backward* sentences and *Nonsense* sentences (the original sentences are used for comparison). First, we examined two **model-derived** measures to determine the degree to which a string supports *syntax-driven sentence-structure building*. We used probability estimates from (i) a lexicalized, probabilistic context-free grammar (PCFG) model (Booth, 1969), and (ii) a powerful neural language model, GPT2-xl (Radford et al., 2019). These two measures are complementary: whereas the PCFG model computes probability estimates based on the structured syntactic representation while ignoring surface-level patterns of word co-occurrence, the GPT2-xl model does the reverse: it computes probability estimates solely based on the surface-level patterns of word co-occurrence, and only implicitly considers information about sentence structure. To derive lexicalized **PCFG surprisal** scores, we use the incremental left-corner parser of Van Schijndel & Schuler (2013), trained on a generalized categorial grammar (Nguyen et al., 2012) reannotation of Wall Street Journal sections 2 through 21 of the Penn Treebank (Marcus et al., 1993) (see also Shain, Blank et al., 2020). To derive a single score per sentence, we obtained a surprisal estimate for each word, and then averaged these estimates across all words in the sentence. The surprisal scores under the **GPT2-xl** model were calculated as the average token surprisal derived from the pre-trained model checkpoint available through the HuggingFace transformers library (Wolf et al., 2020).

Next, we examined a model-derived measure to determine the degree to which a string supports *syntax-independent semantic composition*, i.e., obeys the local semantic dependency structures of naturally occurring language inputs. In other words, this measure quantifies the degree to which a processing algorithm that is syntax-independent might attempt to build complex meanings out of words within a local context based on prior linguistic experience and world knowledge. Following Mollica, Siegelman et al. (2020), we use (positive) Pointwise Mutual Information (**PMI**), an information-theoretic indicator of semantic association (Church & Hanks, 1990), and focus on positive PMI values, because negative values suggest there is no semantic dependency worth building. To derive PPMI scores, we used the procedure described in Mollica, Siegelman et al. (2020) (see Figure SI 4 for a measure of directional PMI). In particular, for each string, we used a sliding four-word window to extract local word pairs (this is equivalent to collecting the bigrams, 1-skip-grams, and 2-skip-grams from each string). For each word pair, we then calculated its PPMI score. Probabilities were estimated using the Google n-gram corpus (Michel et al., 2011) and zs Python library (Smith, 2014) with Laplace smoothing (α=0.1). We obtain a PPMI estimate for each word pair occurring within a four-word sliding window (see Equation (1)), and then average these estimates across all words pairs in the sentence to derive a single score per sentence.


(1) $$PPMI\left(w_{i}\cdots w_{n}\right)=\frac{1}{3\left(n-2\right)}\sum_{i=1}^{n-1}\sum_{j=i+1}^{\min\left(i+3,n\right)}\max\left(0,\log\frac{P\left(w_{i},w_{j}\right)}{P\left(w_{i}\right)P\left(w_{j}\right)}\right)$$

Finally, we evaluated our stimuli in a **behavioral** rating study (see Figure 4A). In particular, we wanted to ensure that our *Nonsense* sentences are judged as grammatically well-formed but lacking conventional meaning by naïve participants. We recruited 120 participants through Amazon’s Mechanical Turk and asked them to rate our *Nonsense* sentence stimuli, along with *Sentence* and *Word list* stimuli for comparison (192 stimuli per condition), for two features on a 1–5 Likert scale: grammatical well-formedness (1=completely ungrammatical to 5=perfectly grammatical) and semantic acceptability (1=doesn’t make any sense to 5=makes perfect sense). As part of the instructions, several examples for each concept were provided. The 576 stimuli were distributed across 4 experimental lists (144 stimuli each, 48 per condition). Each participant completed only one list. In order to counteract poor data quality on MTurk, owing to the use of bots or fake IP addresses (e.g., Chmielewski & Kucker, 2020), we restricted our task to participants with IP addresses in the United States and included only those participants in the analyses who satisfied all of the following criteria: (i) they used the full Likert scale and (ii) they did not, on average, rate *Sentence* stimuli lower than 3 in terms of grammaticality and they did not, on average, rate *Word lists* higher than 3 in terms of grammaticality. Data from 57 participants were included in the final analysis.

#### 2.2.2 Critical task procedure

For our critical fMRI experiment, each experimental list (48 stimuli x 8 conditions = 384 stimuli total) was divided into 8 subsets of 48 stimuli each (6 stimuli per condition), corresponding to 8 scanning runs. A blocked design was used, with each block consisting of 3 trials of the same condition. The trial structure was similar to that used in Mollica, Siegelman et al. (2020): the stimulus (a sequence of 12 words/nonwords) was presented one word/nonword at a time in the center of the screen for 350 ms each, in black capital letters on a white background with no punctuation. The stimulus sequence was followed by a blank screen for 300 ms, then by a memory probe word/nonword presented in blue font for 1,000 ms, and finally, by a blank screen for 500 ms, for a total trial duration of 6 s (thus, experimental blocks were 18 s in duration). When a memory probe appeared, participants were asked to determine whether the probe was the same as the last word/nonword in the sequence they just read, and to indicate their choice via pressing one of two buttons. On half of the trials, the memory probe was the same as the last word/nonword in the sequence; on the remaining trials, probes were randomly sampled correct probes from other stimuli of the same condition from a different block. The memory probe task was designed to be easy and was included to help participants stay alert. Each scanning run (consisting of 16 experimental blocks—2 blocks per condition—and 5 fixation blocks) lasted 16 ∗ 18 s + 5 ∗ 12 s = 348 s (5 min 48s).

#### 2.2.3 Localizers

##### Language network localizer task

The regions of the language network were localized using a task described in detail in Fedorenko et al. (2010) and subsequent studies from the Fedorenko lab (the task is available for download from https://evlab.mit.edu/funcloc/). Briefly, participants silently read sentences and lists of unconnected, pronounceable nonwords (each 12 word-/nonwords-long) in a blocked design. The sentences > nonwords contrast targets brain regions that that support high-level language comprehension. This contrast generalizes across tasks (e.g., Fedorenko et al., 2010; Scott et al., 2017; Ivanova et al., 2020) and presentation modalities (reading vs. listening; e.g. (Fedorenko et al., 2010; Scott et al., 2017; Chen et al., 2021; Malik-Moraleda, Ayyash et al., 2022). All the regions identified by this contrast show sensitivity to lexico-semantic processing (e.g., stronger responses to real words than nonwords) and combinatorial semantic and syntactic processing (e.g. stronger responses to sentences and Jabberwocky sentences than to unstructured word lists and nonword lists) (e.g., Fedorenko et al., 2010, 2016, 2020, 2012; I. Blank et al., 2016; Shain, Kean et al., 2024). More recent work further shows that these regions are also sensitive to sub-lexical regularities (Regev et al., 2024), in line with the idea that this system stores our linguistic knowledge, which encompasses regularities across representational grains, from phonological and morphological schemas to words and constructions (see Fedorenko et al., 2024 for a review). Stimuli were presented one word/nonword at a time at the rate of 450 ms per word/nonword. Participants read the materials passively and performed a simple button-press task at the end of each trial, which was included in order to help participants remain alert. Each participant completed 2 ∼6 min runs.

##### Multiple Demand network localizer task (relevant for some control analyses)

The regions of the Multiple Demand (MD) network (Duncan, 2010; Duncan et al., 2020) were localized using a spatial working memory task contrasting a harder condition with an easier condition (e.g., Fedorenko et al., 2011, 2013; Blank et al., 2014). The hard > easy contrast targets brain regions engaged in cognitively demanding tasks. Fedorenko et al. (2013) have established that the regions activated by this task are also activated by a wide range of other demanding tasks (see also Duncan & Owen, 2000; Hugdahl et al., 2015; Shashidhara et al., 2019; Assem et al., 2020). On each trial (8 s), participants saw a fixation cross for 500 ms, followed by a 3 × 4 grid within which randomly generated locations were sequentially flashed (1 s per flash) 2 at a time for a total of 8 locations (hard condition) or 1 at a time for a total of 4 locations (easy condition). Then, participants indicated their memory for these locations in a 2-alternative, forced-choice paradigm via a button press (the choices were presented for 1,000 ms, and participants had up to 3 s to respond). Feedback, in the form of a green checkmark (correct responses) or a red cross (incorrect responses), was provided for 250 ms, with fixation presented for the remainder of the trial. Hard and easy conditions were presented in a standard blocked design (4 trials in a 32 s block, 6 blocks per condition per run) with a counterbalanced order across runs. Each run included 4 blocks of fixation (16 s each) and lasted a total of 448 s. Each participant completed 2 runs.

#### 2.2.4 Participants

Twenty-two individuals (12 female, 10 male, mean age = 26.3 years, SD = 7.97) were recruited from MIT and the surrounding Cambridge/Boston, MA, community and paid for their participation. All were native speakers of English, had normal hearing and normal or corrected vision, and had no history of language impairment. 20 participants were right-handed, and the remaining 2 were left-handed, as determined by the Edinburgh handedness inventory (Oldfield, 1971), or self-report. All but three participants showed typical left-lateralized activation for the language localizer task (paradigm details above). Lateralization was calculated based on the number of significant (at the fixed whole-brain uncorrected voxel-level threshold of p<0.001) language-responsive voxels in the left vs. right hemispheres (LH vs. RH), using the following formula: $\left(LH-RH\right)/\left(LH+RH\right)$, following Jouravlev et al. (2020). For the three participants, who showed right-lateralized language responses (lateralization values of −0.67, −0.64, and −0.4; individuals with values of −0.25 or below are considered right-lateralized; Jouravlev et al., 2020), we used their right-hemisphere language regions for the analyses. (We show in Figure SI 5 that the results remain unchanged when using the left hemisphere fROIs for all participants.) One participant showed low behavioral performance on the memory probe task (*<*60% accuracy) and was excluded from the analyses, leaving a total of 21 participants. One additional participant showed low behavioral performance for the first 3 runs of the critical task (consistent with self-reported sleepiness); we excluded those runs from the analyses. All participants gave written informed consent in accordance with the requirements of MIT’s Committee on the Use of Humans as Experimental Subjects (COUHES).

#### 2.2.5 fMRI data acquisition, preprocessing, first-level modeling, and fROI definition

##### Data acquisition

Structural and functional data were collected on a whole-body 3 Tesla Siemens Prisma scanner with a 32-channel head coil at the Athinoula A. Martinos Imaging Center at the McGovern Institute for Brain Research at MIT. T1-weighted, Magnetization Prepared Rapid Gradient Echo (MPRAGE) structural images were collected in 208 sagittal slices with 0.85 mm isotropic voxels (TR = 1,800 ms, TE = 2.37 ms, TI = 900 ms, flip = 8 degrees). Functional, blood oxygenation level-dependent (BOLD) data were acquired using an SMS EPI sequence with a 90° flip angle and using a slice acceleration factor of 3, with the following acquisition parameters: seventy-two 2 mm thick near-axial slices acquired in the interleaved order (with 10% distance factor), 2 mm × 2 mm in-plane resolution, FoV in the phase encoding (F >> H) direction 208 mm and matrix size 104 × 104, TR = 2,000 ms, TE = 30 ms, and partial Fourier of 7/8. The first 10 s of each run were excluded to allow for steady state magnetization.

##### Data preprocessing

fMRI data were analyzed using SPM12 (release 7487), CONN EvLab module (release 19b), and other custom MATLAB scripts. Each participant’s functional and structural data were converted from DICOM to NIFTI format. All functional scans were co-registered and resampled using B-spline interpolation to the first scan of the first session (Friston et al., 1995). Potential outlier scans were identified from the resulting subject-motion estimates as well as from BOLD signal indicators using default thresholds in CONN preprocessing pipeline (5 standard deviations above the mean in global BOLD signal change, or framewise displacement values above 0.9 mm; (Nieto-Castañón, 2020). Functional and structural data were independently normalized into a common space (the Montreal Neurological Institute [MNI] template; IXI549Space) using SPM12 unified segmentation and normalization procedure (Ashburner & Friston, 2005) with a reference functional image computed as the mean functional data after realignment across all timepoints omitting outlier scans. The output data were resampled to a common bounding box between MNI-space coordinates (−90, −126, −72) and (90, 90, 108), using 2mm isotropic voxels and 4th order spline interpolation for the functional data, and 1mm isotropic voxels and trilinear interpolation for the structural data. Last, the functional data were smoothed spatially using spatial convolution with a 4 mm FWHM Gaussian kernel.

##### First-level modeling

Effects were estimated using a General Linear Model (GLM) in which each experimental condition was modeled with a boxcar function convolved with the canonical hemodynamic response function (HRF) (fixation was modeled implicitly, such that all timepoints that did not correspond to one of the conditions were assumed to correspond to a fixation period). Temporal autocorrelations in the BOLD signal timeseries were accounted for by a combination of high-pass filtering with a 128 seconds cutoff, and whitening using an AR(0.2) model (first-order autoregressive model linearized around the coefficient a=0.2) to approximate the observed covariance of the functional data in the context of Restricted Maximum Likelihood estimation (ReML). In addition to experimental condition effects, the GLM design included first-order temporal derivatives for each condition (included to model variability in the HRF delays), as well as nuisance regressors to control for the effect of slow linear drifts, subject-motion parameters, and potential outlier scans on the BOLD signal.

##### fROI definition

Following prior work, we used group-constrained, participant-specific functional localization (Fedorenko et al., 2010). Namely, individual activation maps for the target contrast (here, sentences *>* nonwords) were combined with spatial ‘masks’—corresponding to broad areas within which most participants in a large, independent sample show activation for the same contrast. The masks, which were derived in a data-driven way from this independent sample of participants and are available from the lab’s website, have been used in many prior studies (e.g., Diachek, Blank, Siegelman et al., 2020; Jouravlev et al., 2019a; Shain, Blank et al., 2020). They include five regions in each hemisphere: three in the frontal cortex (two in the inferior frontal gyrus, including its orbital portion: IFGorb, IFG; and one in the middle frontal gyrus: MFG), and two in the anterior and posterior temporal cortex (AntTemp and PostTemp). Within each mask, we selected 10% of most localizer-responsive voxels (voxels with the highest t-value for the localizer contrast) following the standard approach in prior work. This approach allows to pool data from the same functional regions across participants while allowing for inter-individual variability in the precise locations of these regions. All main analyses were performed on fMRI BOLD signals extracted from these functional ROIs. For completeness, we also defined a language fROI in the angular gyrus (see Figure SI 6, Table SI 2). This area is activated by the language localizer contrast (e.g., Fedorenko et al., 2010) but has been shown to dissociate from the core frontal and temporal language areas (e.g., Shain, Paunov, Chen et al., 2023).

In addition to the language fROIs, we defined a set of control fROIs using the Multiple Demand network localizer. Here, individual activation maps for the hard > easy contrast were combined with a set of twenty spatial ‘masks’ (10 regions in each hemisphere), which were derived in a data-driven way from an independent sample of participants and are available from the lab’s website. The masks cover the frontal and parietal components of the MD network (Duncan, 2010, 2013) bilaterally. Similar to the language masks, these masks have been used in many prior studies (e.g., Diachek, Blank, Siegelman et al., 2020; Jouravlev et al., 2019a; Shain, Blank et al., 2020). Within each mask, we selected 10% of most localizer-responsive voxels, and the analyses were performed on fMRI BOLD signals extracted from these fROIs.

#### 2.2.5 Estimating responses in the language network to the critical conditions and statistical analyses

**Figure 3.**
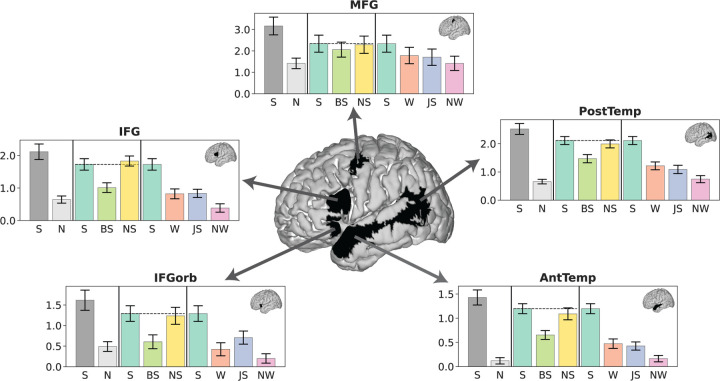
Responses in the five areas of the language network reveal the stability of the observed condition pattern. Neural responses (in % BOLD signal change relative to fixation) to the conditions of the language localizer and critical and control experimental conditions in each of the five language functional regions of interest (fROIs). IFGorb—orbital inferior frontal gyrus, IFG—inferior frontal gyrus, MFG—middle frontal gyrus, AntTemp—anterior temporal lobe, PostTemp—posterior temporal lobe. The profiles of individual fROIs are similar to each other and mirror that of the overall network response (Figure 2D, Table SI 5).

After defining the language fROIs (and, for control analyses, MD fROIs), we extracted the fROIs’ responses to the critical task conditions of interest. To obtain these values, we averaged BOLD responses across voxels within each language fROI in each participant to obtain a value for each condition. Because the fROIs comprising the language network are strongly functionally interconnected (Blank et al., 2014; Mineroff, Blank et al., 2018; Paunov et al., 2019), we perform the key analyses at the network level, but we also show that the response profiles are similar in each individual language fROI (Figure 3).

To evaluate the statistical significance of the differences in the average change in BOLD signal across conditions, we employed a mixed-effect linear regression model with a maximal random-effect structure (Barr et al., 2013), predicting the level of response with a fixed effect and random slope for Condition, and random intercepts for Participants and ROIs (Equation 2).


(2) $${\text{BOLD}}_{\text{system}}∼\text{Condition}+\left(1+\text{Condition|Participant}\right)+\left(1+\text{Condition|ROI}\right)$$

For the analyses at the ROI level, we conducted a mixed-effect linear regression model with a maximal random-effect structure, predicting the level of response with a fixed effect and random slope for Condition, and random intercepts for Participants (Equation 3). In both models, conditions were dummy-coded with the *Sentence* condition as the reference level.

(3) $${\text{BOLD}}_{\text{ROI}}∼\text{Condition}+\left(1+\text{Condition|Participant}\right)$$

## 3. Results

We evaluate the two hypotheses laid out in the Introduction—the syntax-dependent semantic composition hypothesis and the syntax-independent semantic composition hypothesis—across two key analyses. First, we elicit human intuitions about incremental parsing of syntactically ill-formed linguistic inputs using a novel behavioral paradigm. We focus on linguistic inputs that have been previously argued to be processed without a detailed syntactic analysis, based on local semantic cues, and test whether such inputs could instead be reconstructed as they are processed incrementally (Section 3.1). And second, using fMRI, we examine responses in the language brain areas to stimuli designed to adjudicate between the two hypotheses (Section 3.2). In the last section, we briefly examine brain areas that process semantic plausibility, given that we find that this information is not processed within the language areas (Section 3.3).

### 3.1 Word-order scrambled stimuli that elicit a sentence-level response in the language network are amenable to real-time syntactic reconstruction

To investigate whether the invariance of the language network’s response to local word order scrambling (Mollica, Siegelman et al., 2020) may reflect the ability to parse the input following its syntactic reconstruction, rather than shallow, semantics-based comprehension, we probed people’s ability to reconstruct syntactically ill-formed inputs using SynReco—a novel behavioral paradigm (Figure 1A; for a validation of the paradigm, see Figure SI 7). We used the conditions from the Mollica, Siegelman et al.’s study, including intact sentences (*Intact*), sentences with local word order scrambling (with 1, 3, 5, or 7 local swaps; *Scrambled{1,3,5,7}*), and sentences where the word order is scrambled in a way that destroys local dependencies (*Scrambled_LowPMI*) (Figure 1B). We also added a critical new condition, which retains all local dependencies of the *Intact* sentence (Figure 1D) but should make syntactic reconstruction in real time challenging: sentences presented in reverse order (*Backward*).

In the analyses, we focus on the final submitted word orders, i.e., the orders submitted at the last time step. To measure a string’s grammatical well-formedness, we leverage PCFG parser surprisal. PCFG surprisal estimates do not encode surface-level patterns of word co-occurrence directly and instead rely on the structured syntactic representations of stimuli (see also Shain, Blank et al., 2020). To establish a baseline, we first quantified the grammaticality of our set of experimental stimuli across conditions (Figure 1C, hatched bars). We find that, as expected, PCFG surprisal increases numerically with every increase in word-order degradation and is highest for the *Scrambled_LowPMI* and *Backward* conditions (see Table SI 3, **Experimental Items**, for pairwise Tukey’s HSD (honestly significant difference) significance testing).

Next, we quantified the grammaticality of participants’ reconstructions of these stimuli across conditions (Figure 1C, solid bars). We find that for all conditions where word order was perturbed, participants managed to make the stimuli more grammatically well-formed (Figure 1C, D), even though every additional word swap was associated with a significant decrease in the ability to reconstruct the original sentence verbatim (see Figure 1E, Table SI 4), consistent with the offline reconstruction results reported in Mollica, Siegelman et al. (2020). Critically, reconstructions of stimuli with local word order swaps, for which Mollica, Siegelman et al. report sentence-level responses in the language network (*Scrambled{1,3,5,7}*), mostly do not significantly differ from one another (see Table SI 3 for all pairwise comparisons) but are all significantly more well-formed than reconstructions of the *Scrambled_LowPMI* stimuli where local dependencies are destroyed and for which responses in the language network are low (all ps < 0.001, Table SI 3). In other words, this paradigm effectively captures behavioral patterns that plausibly underlie the observed pattern of responses in the language areas, as reported by Mollica, Siegelman et al. (2020). The stimuli in the *Backward* condition pattern with the stimuli in the *Scrambled_LowPMI* condition (Table SI 3). If the ability to parse the input (following incremental reconstruction, if need be, for syntactically ill-formed inputs) is indeed tied to the level of response to those inputs in the language brain areas, we would expect the *Backward* condition to pattern with the *Scrambled_LowPMI* condition (i.e., to elicit a relatively low response). This is indeed what we find, as discussed next.

### 3.2 Syntax-driven, not syntax-independent, semantic composition drives the language network’s response

In Section 3.1, we established that stimuli with local word order scrambling that have previously been shown to elicit a sentence-level response in the language network are amenable to real-time syntactic reconstruction, whereas stimuli with more severe word order scrambling are not. One of the latter conditions, the *Scrambled_LowPMI* condition, where local dependencies are destroyed, was shown by Mollica, Siegelman et al. (2020) to elicit a relatively low response in the language network. The *Backward* condition is similar to the *Scrambled_LowPMI* condition in the difficulty of online syntactic reconstruction but importantly, local dependencies are preserved. This condition therefore constitutes a critical test for the syntax-independent semantic composition hypothesis: if the language network’s response is driven by shallow/associative processing operations, which are guided by local, word-order-independent semantic relationships, then this condition should elicit a strong response, similar to these brain regions’ response to well-formed sentences (see Figure 2C for quantitative evidence of similar local combinability; see Section 2.2.1 for details of this measure). If, on the other hand, language comprehension is syntax-driven, then this condition should elicit a low response in the language network given that syntactic structure cannot be reconstructed incrementally and thus the input cannot be parsed (see also Figure 2C for quantitative evidence of lower syntactic well-formedness based on PCFG surprisal; see Section 2.2.1 for details of this measure).

Another condition that helps distinguish between our two hypotheses are grammatically well-formed sentences where building complex meanings is impeded by semantic implausibility (*Nonsense* sentences). If language comprehension is syntax-driven, this condition should elicit a strong response in the language network (see Figure 2C, leftmost panel, for quantitative evidence of syntactic well-formedness based on PCFG surprisal); in contrast, if language comprehension is shallow/associative, then this condition should elicit a low response because the lack of plausible semantic dependencies should discourage complex meaning construction (see Figure 2C, rightmost, for quantitative evidence of local combinability based on PPMI).

In fMRI, we collected responses to *Backward* sentences and *Nonsense* sentences, as well as to well-formed sentences and three linguistic control conditions that are commonly used to probe computations related to lexical access vs. structure building and that can be helpful for interpreting the responses to the critical conditions—*Word Lists*, *Jabberwocky Sentences,* and *Nonword Lists* (e.g., Fedorenko et al., 2010, 2012, 2016; Shain, Kean et al., 2024).

Condition-level effects in the language network are reported in Figure 2D. First, replicating much prior work (e.g., Fedorenko et al., 2010, 2012, 2016; Shain, Kean et al., 2024), we found a pattern whereby the *Intact Plausible Sentence (Sentence)* condition elicited the strongest response in the language areas, *Word Lists* and *Jabberwocky Sentences* elicited a lower response, and *Nonword Lists* elicited the lowest response (Figure 2D, control conditions). Second and critically, we found that the *Backward Sentence* condition elicited a significantly lower response relative to the *Sentence* condition, whereas the *Nonsense Sentence* condition elicited a response that was similar in magnitude to and statistically indistinguishable from that observed for *Sentences* (Table 1, Figure 2D, critical conditions; see Figure SI 5 for evidence that the results hold when using left-hemisphere language fROIs for all participants, including those with right-lateralized language activations). The results also held—both qualitatively and statistically—for each language ROI separately (Figure 3 and Table SI 5), and in individual participants (Figure SI 8), evidencing their robustness. (Even if the small numerical difference in magnitude between the language network’s response to *Sentence* vs. *Nonsense Sentence* conditions—driven by the two temporal fROIs (see Figure 3)—becomes statistically significant in a larger dataset, this difference is too small to be practically significant (Sullivan & Feinn, 2012): the *Nonsense Sentence* condition’s magnitude is ~97% of the *Sentence* condition’s magnitude; cf. the *Backward Sentence* condition, which is only ~67% of the *Sentence* condition’s magnitude.)

The observed pattern of neural responses challenges the claim from Mollica, Siegelman et al. (2020) that the ability to form dependencies among nearby words is necessary and sufficient to elicit a sentence-level BOLD response in the language network. In particular, the network’s strong response to *Nonsense* sentences suggests that local combinability is *not necessary* to drive the network (or, at least, that the critical aspects of local combinability have to do with *syntax* rather than meaning); and the network’s low response to *Backward* sentences suggests that local combinability is *not sufficient* to elicit a sentence-level response. Instead, the findings support the hypothesis whereby the language network supports syntax-dependent semantic composition.

### 3.3 Semantic plausibility is evaluated outside of the language network

**Figure 4.**
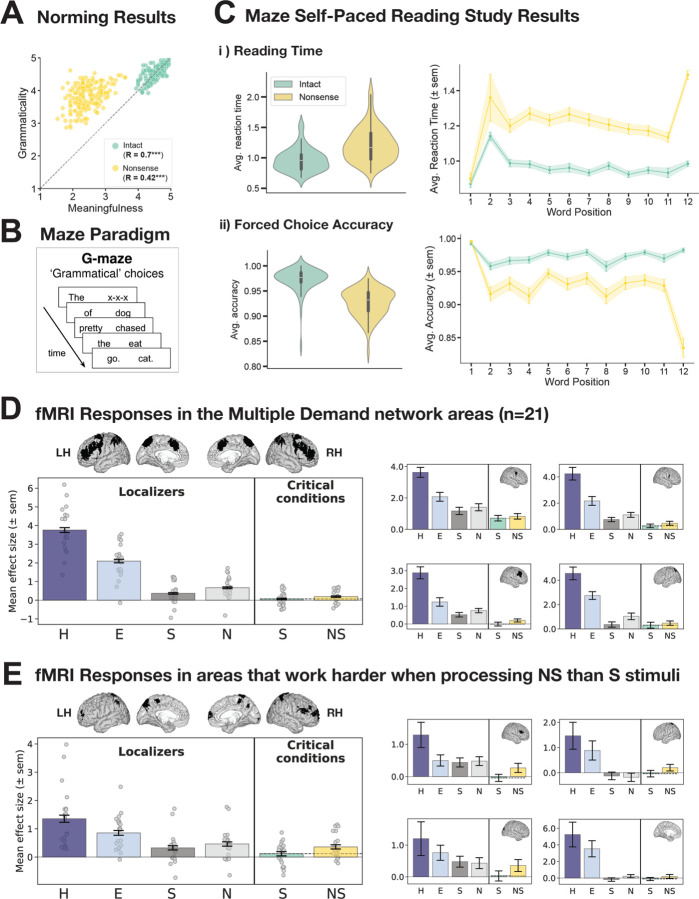
Semantic plausibility is processed by brain regions outside of the language network. Panels A-E focus on the cost associated with semantic implausibility; Panel F focuses on brain regions that respond more to plausible sentences. **A)** Behavioral norming study results for *Sentence* and *Nonsense* sentence stimuli (for details, see Section 2.2.1; for the full set of results, including *Word list* stimuli, see Figure SI 9). *Nonsense* sentences are rated as less meaningful, but also less grammatical than typical sentences (likely because human judgments of sentence grammaticality and meaningfulness tend to be correlated). **B)** Illustration of the Maze experimental paradigm. **C)** Maze study results for reading times (top row plots) and forced choice accuracy (bottom row plots). Violin plots show average across all positions, line plots show averages per word position. Processing *Nonsense* sentences is measurably more costly than processing typical sentences. **D)** Neural responses (in % BOLD signal change relative to fixation) to the conditions of the Multiple Demand (MD) and language localizer tasks and the *Sentence* and *Nonsense sentence* conditions of the critical experiment in the MD network. For responses to all experimental conditions and statistical analysis see Figure SI 10, Table SI 6. The black masks on the brain show the parcels that were used to define the MD areas in individual participants (see Section 2.2.3 for details). The large plot shows the average response profile across the MD network. Dots show individual subject responses; error bars show standard errors of the mean by participants. The smaller plots show the response of four sample MD ROIs; inset brains show the location of the respective parcel. The MD network as a whole shows a numerically, albeit not significantly, higher response to *Nonsense* sentences than typical sentences. **E)** Neural responses (in % BOLD signal change relative to fixation) to the conditions of the Multiple Demand (MD) and language localizer tasks and the *Sentence* and *Nonsense sentence* conditions of the critical experiment averaged across brain areas derived from a whole-brain search for areas that respond to the *Nonsense sentence* > *Sentence* contrast (n=9 regions total; responses are estimated with across-runs cross-validation) (see Figure SI 11 for details). Dots show individual subject responses; error bars show standard errors of the mean by participants. These regions and their responses to the conditions of the MD network localizer suggest that they constitute a subset of the MD network. **F)** Neural responses (in % BOLD signal change relative to fixation) to the conditions of the Multiple Demand (MD) and language localizer tasks and the *Sentence* and *Nonsense sentence* conditions of the critical experiment averaged across brain areas derived from a whole-brain search for areas that respond to the *Sentence* > *Nonsense sentence* contrast (n=14 regions total; responses are estimated with across-runs cross-validation) (see Figure SI 12 for details). Dots show individual subject responses; error bars show standard errors of the mean by participants. These regions and their responses to the conditions of the MD network localizer suggest that they constitute a subset of the Default Mode network.

In Section 3.2, we established that *Nonsense* sentences elicit a BOLD response in the language network that is similar in magnitude to that elicited by meaningful sentences, which suggests that this particular brain system is relatively insensitive to semantic plausibility information (Figure 2D). However, behaviorally, *Nonsense* sentences are rated as less meaningful and less grammatical than typical plausible sentences (Figure 4A), so there must be a cost to processing them (see Marslen-Wilson & Tyler, 1975, 1980 for earlier evidence). In an effort to understand these effects better, we quantified this cost using self-paced reading estimates and searched for brain regions outside of the language network that are sensitive to semantic plausibility.

We used Boyce et al.’s (2020) version of the Maze self-paced reading paradigm (Freedman and Forster, 1985; Forster et al., 2009), where participants read stimuli word-by-word by successively choosing the likely next word over a contextually inappropriate distractor word in a forced-choice design (Figure 4B; Boyce et al., 2020; Wilcox et al., 2021) (for experiment details see Supplementary Methods). We find that processing *Nonsense* sentences is associated with significantly longer reading times (paired-samples t-test, *Nonsense* vs. *Sentence*: t=14.3; p<0.001) and significantly lower choice accuracy (paired-samples t-test, *Nonsense* vs. *Sentence*: t=−18.72; p<0.001) (Figure 4C, violin plots). Furthermore, the processing cost is immediate and persistent, manifesting across all word positions where a distractor word is present (i.e., starting with the second word), with the highest processing cost observed at the final position, i.e., at the word that co-occurred with a sentence-final period (Figure 4C, line plots).

What brain system might process the cost associated with semantic implausibility during language comprehension? First, we examined the responses to our critical conditions within the domain-general Multiple Demand (MD) network, which is recruited whenever humans solve demanding cognitive tasks (Duncan, 2010, 2013; Duncan et al., 2020). Although this network does not contribute to core linguistic computations related to lexical access and syntactic structure building for naturalistic linguistic inputs, in the absence of external task demands (Diachek, Blank, Siegelman et al., 2020; Shain, Blank et al., 2020; see Fedorenko & Shain, 2021 for review), it has been implicated in the processing of some perceptually and linguistically degraded linguistic inputs (e.g., Fedorenko, 2014; Kuperberg et al., 2003). For example, much prior work has reported stronger responses in this network to word lists and nonword lists than to sentences (Mineroff, Blank et al., 2018; Diachek, Blank, Siegelman et al., 2020), and Mollica, Siegelman et al. (2020) found that some regions within this network exhibit an increase in activity for degraded-word-order stimuli. We replicate these effects—for both the language localizer conditions and the conditions of the critical experiment—and extend them to a new condition (sentences presented backwards) (Figure SI8). For our critical contrast, between *Nonsense* and typical, plausible *Sentences*, the MD network as a whole showed a small numerically, albeit not significantly, higher response to *Nonsense* sentences (Figure 4D, large plot; Table SI 6); a few regions, mostly in the right hemisphere, showed this effect most clearly, although the magnitude of the effect is small even in those regions (Figure 4D, small plots).

To test whether areas elsewhere in the brain may show a higher response to *Nonsense* sentences, we additionally performed a group-constrained subject-specific (GSS) whole-brain analysis (Fedorenko et al., 2010; Julian et al., 2012). This analysis is similar to the standard random-effects group analysis in fMRI but allows for inter-individual variability in the precise locations of functional areas, which is known to exist in the association cortex (Frost & Goebel, 2012; Tahmasebi et al., 2012), yielding higher sensitivity (Nieto-Castañón & Fedorenko, 2012). We found a few areas where most participants showed a small *Nonsense sentences > Plausible sentences* effect (quantified with an across-runs cross-validation procedure); however, the topography of these regions and their responses to the MD network localizer conditions suggest that they constitute a subset of the MD network (Figure 4E; Figure SI 11). Thus, the cognitive cost associated with the processing of semantically implausible sentences is carried, in a distributed fashion, by parts of the domain-general MD network.

In addition to brain regions that are sensitive to the cost of semantic implausibility, there must also exist brain regions that respond more when the sentences convey plausible meanings that can be related to our general world knowledge. Therefore, we also performed a GSS whole-brain analysis for the *Sentences* > *Nonsense sentences* contrast. This search yielded a few regions that showed reliably greater responses to this contrast (quantified with across-runs cross-validation). Some of these regions lie in close spatial proximity to the language network, but their profiles are clearly functionally distinct (Figure 4F; Figure SI 12; see Ivanova, 2022 for related evidence). In particular, these regions resemble the profile of the Default Mode network (Buckner & DiNicola, 2019): they deactivate to the demanding spatial working memory task (and more so for the more demanding condition) and they respond weakly or not at all to the language localizer contrast (e.g., Mineroff, Blank et al., 2018; Braga et al., 2020; DiNicola et al., 2023). These findings align with claims that the Default Mode network supports aspects of semantic processing (Binder et al., 1997; Wirth et al., 2011; Jackson et al., 2016; Baldassano et al., 2018).

## 4. Discussion

Language inputs typically adhere to grammatical rules and express propositions that align with our knowledge of the world. But to what degree does comprehension rely on syntactic vs. semantic cues? We pushed the grammaticality and meaningfulness of linguistic inputs to their logical extremes—obliterating parsability or meaningfulness—and found support for strong reliance on the syntactic mode of processing, rather than on shallow/semantics-based processing, in the language network. Below, we discuss these findings further and contextualize them with respect to past work.

### 4.1 The language network’s core computation is syntax-dependent semantic composition

We found that the language network is engaged *whenever parsing is possible,* either on the stimuli in their original form, or following reconstruction. Mollica, Siegelman et al. (2020) reported a relative insensitivity of the language network’s (Fedorenko et al., 2024) response to local word-order scrambling. They attempted to rule out a reconstruction interpretation, but their reconstruction task may not provide a good measure of *real-time interpretability*. Indeed, using an incremental reconstruction task, we found that the locally-scrambled sentences are amenable to real-time reconstruction. Thus, the relative insensitivity of the language network to local scrambling plausibly reflects the parsability of these stimuli following incremental reconstruction of the sentence structure, not the fact that they are processed in a shallow way, based on semantic cues.

We further found that sentences presented backwards pose difficulty for real-time reconstruction, and elicit a low response in the language areas. Thus, the local combinability of words—which is preserved in these stimuli—does not invariably lead to a sentence-level response in the language areas, contra Mollica, Siegelman et al. (2020). Without syntactic (e.g., word order) cues, our prior linguistic experience and world knowledge cannot effectively guide interpretation. At the same time, we found that nonsensical sentences elicit as high a response in the language areas as plausible sentences in spite of the fact that our prior linguistic experience and world knowledge should guide our parser away from attempts to build syntactic structure. We therefore conclude that the critical aspects of local combinability that the language network cares about have to do with *syntactic* combinatoriality.

### 4.2 Syntactic reconstruction is supported by the domain-general Multiple Demand network

The language areas responded weakly to backward sentences; in contrast, the areas of the domain-general Multiple Demand (MD) network (Duncan, 2010, 2013; Duncan et al., 2020) responded more strongly to backward sentences than to well-formed sentences (**SI-9**). Together with Mollica, Siegelman et al.’s (2020) findings, these results suggest that syntactic reconstruction costs are carried by the MD network, which supports diverse computations during cognitively demanding tasks (Duncan & Owen, 2000; Fedorenko et al., 2013; Hugdahl et al., 2015; Shashidhara et al., 2019; Assem et al., 2020). Thus, although the MD network does not appear to support computations related to lexical access and syntactic parsing for well-formed linguistic inputs (e.g., Diachek, Blank, Siegelman et al., 2020; see Fedorenko & Shain, 2021 for review), it evidently supports syntactic reconstruction of corrupt inputs. Syntactically corrupt language stimuli may therefore provide a fertile testbed for probing inter-network interactions using temporally-resolved methods, like intracranial recordings. For example, the MD network must show strong stimulus-related activity for stimuli that require reconstruction (cf. Blank & Fedorenko, 2017), and it must be engaged early on, or even simultaneously with the language network given the speed of language comprehension.

### 4.3 Syntax versus other information sources during language comprehension

The similarly strong response of the language network to plausible sentences and grammatical strings that do not express conventional meanings aligns with the human ability to understand novel sentences, including those that describe unusual events or scenarios that are hypothetical, counterfactual, or fictitious. This is the generative power of language: our language system must be able to apply its computations to any syntactically well-formed sequence of words. Nevertheless, language statistics also reflect the distributional properties of objects and events in the world (Mikolov et al., 2013; Pennington et al., 2014; Roads & Love, 2020; Abdou et al., 2021; Kauf, Ivanova et al., 2023), which might lead to the expectation of sensitivity of the language brain areas to the plausibility of sentence meanings. Furthermore, a large body of psycholinguistic work has demonstrated that language comprehension is affected by diverse information sources other than syntax (e.g., MacDonald et al., 1994; Tanenhaus et al., 1995), including world knowledge (e.g., Hagoort et al., 2004; McRae & Matsuki, 2009).

Debates about whether syntactic information is processed separately from other information that can affect language comprehension raged in the psycholinguistic literature in the 1980s-2000s (for summaries, see e.g., Gibson & Pearlmutter, 1998; Clifton Jr. et al., 2003). Recent advances in our understanding of the neural architecture of language allow us to revisit these questions through a new lens. In particular, given the separability of the language-selective network from other brain systems (Fedorenko et al., 2024), this question can be recast as *whether non-syntactic information gets represented and processed in the language network*. We have already learned for example, that in line with the behavioral evidence of quick integration of lexical cues during interpretation (e.g., MacDonald et al., 1994), there doesn’t appear to be spatial segregation between neural populations that process syntactic structure and those that process word meanings (e.g., Fedorenko et al., 2020; Hu, Small et al., 2023; Shain, Kean et al., 2024). In contrast, information like gestures, facial expressions, and prosody appear to be processed by brain areas that are distinct from the language network (e.g., Deen et al., 2015; Pritchett et al., 2018; Jouravlev et al., 2019b; Regev et al., in prep.). Similarly, discourse-level structure appears to be processed in distinct, non-language-selective systems (Ferstl & von Cramon, 2001; Kuperberg et al., 2006; Ferstl et al., 2008; Lerner et al., 2011; Jacoby & Fedorenko, 2020; see Fedorenko et al., 2024 for a review).

What about world knowledge/plausibility? The fact that sentence plausibility does not affect the computations of the language network suggests that distinct brain systems support linguistic decoding vs. evaluating the meanings with respect to world knowledge. Prior evidence supports a dissociation between linguistic and general-semantic processing: pre-verbal infants and individuals with aphasia (linguistic deficits) can understand the world (e.g., Hirsh-Pasek & Golinkoff, 2010; Spelke, 2023; Chertkow et al., 1997; Saygın et al., 2004; Antonucci & Reilly, 2008; Warren & Dickey, 2021) and make sophisticated judgments about objects and events (e.g., Varley & Siegal, 2000; Dickey & Warren, 2015; Colvin et al., 2019; Ivanova et al., 2021; Benn, Ivanova et al., 2023), and distinct brain areas are activated by i) linguistic event descriptions selectively versus ii) both linguistic and non-linguistic events (Baldassano et al., 2018; Wurm & Caramazza, 2019; Ivanova et al., 2021). In line with the latter, we find that sentence plausibility is processed in brain regions that are distinct from the language-selective regions, although located in proximity to them (Figure 4F; Figure SI 12; see Ivanova et al., 2022 for related evidence).

Spatial separability between language areas and semantic areas (or areas that process eye gaze and gestures) need not imply temporal staging of linguistic decoding vs. the processing of non-linguistic information. Many brain areas may work in parallel and exchange information on a fast timescale during incremental comprehension (although the details of these parallel computations and inter-areal/inter-network interactions remain poorly understood). However, it is also possible that—at least with respect to world knowledge—some temporal staging is required: after all, to understand *that* a sentence is implausible, you need to first decode its meaning.

### 4.4 Limitations, future directions, and open questions

None of the current methods in language research directly tap into the parsing operations that humans engage in. The behavioral paradigm we developed to elicit incremental intuitions about parsing is a step in the right direction, but future work should better align the timing of the task with real-life comprehension, incorporate working memory constraints (Christiansen & Chater, 2016), and expand the repertoire of reconstruction operations (Gibson et al., 2013). Future work should also test whether the current results generalize to flexible-word-order languages, and investigate comprehension of syntactically degraded stimuli in longer contexts given past evidence of contextual influences (e.g., Spivey & Tanenhaus, 1998; Chen et al., 2023).

Interpretation-wise, we discuss our results in terms of syntax-driven semantic composition, but they could alternatively be construed in terms of prediction (e.g., Kuperberg & Jaeger, 2016). In other words, perhaps the language network gets engaged whenever we process linguistic stimuli that are predictable to some degree. The strong response to *Nonsense* sentences rules out a version of this hypothesis that has to do with *overall* predictability (Figures 2–3; see Figure SI 13 for additional evidence), but our results are compatible with *purely syntactic* predictability. In fact, it is unclear whether syntactic integration and syntactic predictability could even in principle be distinguished given that stimuli where syntactic integration is possible are necessarily characterized by some degree of syntactic predictability.

Future studies should further illuminate the contributions of the Multiple Demand (MD) network to syntactic reconstruction. For example, these findings could be connected to the ERP research that has interpreted the domain-general P600 component (e.g., Osterhout & Holcomb, 1992; Patel, 2003; Núñez-Peña & Honrubia-Serrano, 2004; Cohn et al., 2012) as indexing error correction (Ryskin, Stearns et al., 2021). Our findings also make predictions about the processing of syntactically corrupt inputs in children and older adults (given that the MD network is slow to develop (Fiske & Holmboe, 2019; Schettini et al., 2023) and shows clear age-related decline (Reuter-Lorenz et al., 2000; Mitchell et al., 2023; Wu & Hoffman, 2023)). If the MD network cannot effectively support syntactic reconstruction in these populations, we should observe larger effects of syntactic degradation on the language network’s responses, and greater reliance on semantic plausibility in interpreting corrupt inputs (e.g., Beese et al., 2019).

Future work should also investigate a) the time-course and nature of the language network’s interaction with non-language-specific systems during incremental comprehension; b) the criteria that determine whether a stimulus is in-domain vs. out-of-domain for the language network (e.g., a locally scrambled sentence vs. a list of unconnected words) (see Shain, Kean et al., 2024 for further discussion); and c) the nature of linguistic representations and computations. Regarding the latter, we have here talked about parsing in terms of symbolic operations (Pullum & Gazdar, 1982; Joshi, 1985; Steedman, 2000; Chomsky, 2014), but neural network language models suggest that explicit encoding of symbolic components may not be necessary: these models reliably encode sentence structure in their embeddings (e.g., Hewitt & Manning, 2019; Sinha et al., 2021; Tucker et al., 2021; Eisape et al., 2022; see Pavlick, 2023 for discussion). Whether syntactic representations in the human brain might similarly be encoded without explicit symbolic representations, and whether/how symbolic-like representations emerge in neural network architectures remain exciting questions for future work.

## Supplementary Material

Supplement 1

## Figures and Tables

**Table 1:** Results of mixed-effects linear regression for fMRI responses within the language network. Stimulus type was dummy-coded with *Intact sentence* as the reference level.

	Estimate	Est. error	95% CI	
Intact sentence	1.45*	0.53	0.43	2.54
Backward sentence vs. Intact sentence	−0.55*	0.13	−0.80	−0.30
Nonsense sentence vs. Intact sentence	−0.08	0.15	−0.38	0.22
Word list vs. Intact sentence	−0.77*	0.13	−1.02	−0.52
Jabberwocky sentence vs. Intact sentence	−0.75*	0.13	−1.00	−0.50
Nonword list vs. Intact sentence	−1.09*	0.14	−1.36	−0.82

* Denotes significant difference.

## Data Availability

Data and code will be made publicly available upon publication.

## Significance statement

Whether language comprehension relies predominantly on structural (syntactic) cues or meaning-related (semantic) cues remains debated. We shed new light on this question by examining the language brain areas’ responses to stimuli where syntactic and semantic cues are pitted against each other, using fMRI. We find that the language areas respond weakly to stimuli that allow for local semantic composition but cannot be parsed syntactically—as confirmed in a novel behavioral paradigm—and they respond strongly to grammatical but semantically implausible sentences, like the famous ‘Colorless green ideas sleep furiously’ sentence. These findings challenge accounts of language processing that suggest that syntactic parsing can be foregone in favor of shallow semantic processing.

## References

[R1] Abdou M., Kulmizev A., Hershcovich D., Frank S., Pavlick E., & Søgaard A. (2021). Can Language Models Encode Perceptual Structure Without Grounding? A Case Study in Color. Proceedings of the 25th Conference on Computational Natural Language Learning, 109–132.

[R2] Antonucci S. M., & Reilly J. (2008). Semantic memory and language processing: A primer. Seminars in Speech and Language, 29(01), 005–017. 10.1055/s-2008-1061621 18348088

[R3] Ashburner J., & Friston K. J. (2005). Unified segmentation. Neuroimage, 26(3), 839–851. 15955494 10.1016/j.neuroimage.2005.02.018

[R4] Assem M., Glasser M. F., Van Essen D. C., & Duncan J. (2020). A domain-general cognitive core defined in multimodally parcellated human cortex. Cerebral Cortex, 30(8), 4361– 4380. 32244253 10.1093/cercor/bhaa023 PMC7325801

[R5] Baldassano C., Hasson U., & Norman K. A. (2018). Representation of real-world event schemas during narrative perception. Journal of Neuroscience, 38(45), 9689–9699. 30249790 10.1523/JNEUROSCI.0251-18.2018 PMC6222059

[R6] Beese C., Werkle-Bergner M., Lindenberger U., Friederici A. D., & Meyer L. (2019). Adult age differences in the benefit of syntactic and semantic constraints for sentence processing. Psychology and Aging, 34(1), 43. 30284854 10.1037/pag0000300

[R7] Benn Y., Ivanova A. A., Clark O., Mineroff Z., Seikus C., Silva J. S., Varley R., & Fedorenko E. (2023). The language network is not engaged in object categorization. Cerebral Cortex, 33(19), 10380–10400. 37557910 10.1093/cercor/bhad289 PMC10545444

[R8] Ben-Shachar M., Hendler T., Kahn I., Ben-Bashat D., & Grodzinsky Y. (2003). The neural reality of syntactic transformations: Evidence from functional magnetic resonance imaging. Psychological Science, 14(5), 433–440. 12930473 10.1111/1467-9280.01459

[R9] Bicknell K., Elman J. L., Hare M., McRae K., & Kutas M. (2010). Effects of event knowledge in processing verbal arguments. Journal of Memory and Language, 63(4), 489–505. 21076629 10.1016/j.jml.2010.08.004 PMC2976562

[R10] Binder J. R., Frost J. A., Hammeke T. A., Cox R. W., Rao S. M., & Prieto T. (1997). Human brain language areas identified by functional magnetic resonance imaging. The Journal of Neuroscience: The Official Journal of the Society for Neuroscience, 17(1), Article 1. 10.1523/JNEUROSCI.17-01-00353.1997 PMC6793702 8987760

[R11] Blank I. A., & Fedorenko E. (2017). Domain-General Brain Regions Do Not Track Linguistic Input as Closely as Language-Selective Regions. Journal of Neuroscience, 37(41), Article 41. 10.1523/JNEUROSCI.3642-16.2017 PMC5637122 28871034

[R12] Blank I., Balewski Z., Mahowald K., & Fedorenko E. (2016). Syntactic processing is distributed across the language system. Neuroimage, 127, 307–323. 26666896 10.1016/j.neuroimage.2015.11.069 PMC4755877

[R13] Blank I., Kanwisher N., & Fedorenko E. (2014). A functional dissociation between language and multiple-demand systems revealed in patterns of BOLD signal fluctuations. Journal of Neurophysiology, 112(5), 1105–1118. 24872535 10.1152/jn.00884.2013 PMC4122731

[R14] Booth T. L. (1969). Probabilistic representation of formal languages. 10th Annual Symposium on Switching and Automata Theory (Swat 1969), 74–81.

[R15] Boyce V., Futrell R., & Levy R. P. (2020). Maze Made Easy: Better and easier measurement of incremental processing difficulty. Journal of Memory and Language, 111, 104082.

[R16] Braga R. M., DiNicola L. M., Becker H. C., & Buckner R. L. (2020). Situating the left-lateralized language network in the broader organization of multiple specialized large-scale distributed networks. Journal of Neurophysiology, 124(5), Article 5. 10.1152/jn.00753.2019 PMC8356783 32965153

[R17] Buckner R. L., & DiNicola L. M. (2019). The brain’s default network: Updated anatomy, physiology and evolving insights. Nature Reviews Neuroscience, 20(10), 593–608. 31492945 10.1038/s41583-019-0212-7

[R18] Carroll L. (1872). Jabberwocky. Through the Looking Glass and What Alice Found There.

[R19] Chen S., Nathaniel S., Ryskin R., & Gibson E. (2023). The effect of context on noisy-channel sentence comprehension. Cognition, 238, 105503. 37302257 10.1016/j.cognition.2023.105503 PMC11729398

[R20] Chen X., Affourtit J., Ryskin R., Regev T. I., Norman-Haignere S., Jouravlev O., Malik-Moraleda S., Kean H., Varley R., & Fedorenko E. (2021). *The human language system does not support music processing* (p. 2021.06.01.446439). bioRxiv. 10.1101/2021.06.01.446439 PMC10505454 37005063

[R21] Chertkow H., Bub D., Deaudon C., & Whitehead V. (1997). On the status of object concepts in aphasia. Brain and Language, 58(2), 203–232. 9182748 10.1006/brln.1997.1771

[R22] Chmielewski M., & Kucker S. C. (2020). An MTurk crisis? Shifts in data quality and the impact on study results. Social Psychological and Personality Science, 11(4), 464–473.

[R23] Chomsky N. (1957). Syntactic structures. Mouton. Chomsky, N. (2014). The minimalist program. MIT press.

[R24] Christiansen M. H., & Chater N. (2016). The Now-or-Never bottleneck: A fundamental constraint on language. Behavioral and Brain Sciences, 39, e62. 10.1017/S0140525X1500031X 25869618

[R25] Church K., & Hanks P. (1990). Word association norms, mutual information, and lexicography. Computational Linguistics, 16(1), 22–29.

[R26] Clifton C. Jr, Traxler M. J., Mohamed M. T., Williams R. S., Morris R. K., & Rayner K. (2003). The use of thematic role information in parsing: Syntactic processing autonomy revisited. Journal of Memory and Language, 49(3), 317–334.

[R27] Cohn N., Paczynski M., Jackendoff R., Holcomb P. J., & Kuperberg G. R. (2012). (Pea) nuts and bolts of visual narrative: Structure and meaning in sequential image comprehension. Cognitive Psychology, 65(1), 1–38. 22387723 10.1016/j.cogpsych.2012.01.003 PMC3331971

[R28] Colvin M., Warren T., & Dickey M. W. (2019). Event knowledge and verb knowledge predict sensitivity to different aspects of semantic anomalies in aphasia. Grammatical Approaches to Language Processing: Essays in Honor of Lyn Frazier, 241–259.

[R29] Constable R. T., Pugh K. R., Berroya E., Mencl W. E., Westerveld M., Ni W., & Shankweiler D. (2004). Sentence complexity and input modality effects in sentence comprehension: An fMRI study. NeuroImage, 22(1), 11–21. 15109993 10.1016/j.neuroimage.2004.01.001

[R30] De Vincenzi M., Job R., Di Matteo R., Angrilli A., Penolazzi B., Ciccarelli L., & Vespignani F. (2003). Differences in the perception and time course of syntactic and semantic violations. Brain and Language, 85(2), 280–296. 12735945 10.1016/s0093-934x(03)00055-5

[R31] Deen B., Koldewyn K., Kanwisher N., & Saxe R. (2015). Functional organization of social perception and cognition in the superior temporal sulcus. Cerebral Cortex, 25(11), 4596– 4609. 26048954 10.1093/cercor/bhv111 PMC4816802

[R32] Demberg V., & Keller F. (2008). Data from eye-tracking corpora as evidence for theories of syntactic processing complexity. Cognition, 109(2), Article 2. 10.1016/j.cognition.2008.07.008 18930455

[R33] Diachek E., Blank I., Siegelman M., Affourtit J., & Fedorenko E. (2020). The domain-general multiple demand (MD) network does not support core aspects of language comprehension: A large-scale fMRI investigation. Journal of Neuroscience, 40(23), 4536– 4550. 32317387 10.1523/JNEUROSCI.2036-19.2020 PMC7275862

[R34] Dickey M. W., & Warren T. (2015). The influence of event-related knowledge on verb-argument processing in aphasia. Neuropsychologia, 67, 63–81. 25484306 10.1016/j.neuropsychologia.2014.12.003 PMC4297691

[R35] DiNicola L. M., Sun W., & Buckner R. L. (2023). Side-by-side regions in dorsolateral prefrontal cortex estimated within the individual respond differentially to domain-specific and domain-flexible processes. Journal of Neurophysiology, 130(6), 1602–1615. 37937340 10.1152/jn.00277.2023 PMC11068361

[R36] Ditman T., Holcomb P. J., & Kuperberg G. R. (2007). An investigation of concurrent ERP and self-paced reading methodologies. Psychophysiology, 44(6), 927–935. 17850242 10.1111/j.1469-8986.2007.00593.x PMC2692571

[R37] Duncan J. (2010). The multiple-demand (MD) system of the primate brain: Mental programs for intelligent behaviour. Trends in Cognitive Sciences, 14(4), Article 4. 10.1016/j.tics.2010.01.004 20171926

[R38] Duncan J. (2013). The structure of cognition: Attentional episodes in mind and brain. Neuron, 80(1), 35–50. 24094101 10.1016/j.neuron.2013.09.015 PMC3791406

[R39] Duncan J., Assem M., & Shashidhara S. (2020). Integrated intelligence from distributed brain activity. Trends in Cognitive Sciences, 24(10), 838–852. 32771330 10.1016/j.tics.2020.06.012 PMC7116395

[R40] Duncan J., & Owen A. M. (2000). Common regions of the human frontal lobe recruited by diverse cognitive demands. Trends in Neurosciences, 23(10), 475–483. 11006464 10.1016/s0166-2236(00)01633-7

[R41] Eisape T., Gangireddy V., Levy R., & Kim Y. (2022). Probing for Incremental Parse States in Autoregressive Language Models. Findings of the Association for Computational Linguistics: EMNLP 2022, 2801–2813.

[R42] Erickson T. D., & Mattson M. E. (1981). From words to meaning: A semantic illusion. Journal of Verbal Learning and Verbal Behavior, 20(5), 540–551.

[R43] Federmeier K. D., & Kutas M. (1999). A rose by any other name: Long-term memory structure and sentence processing. Journal of Memory and Language, 41(4), 469–495.

[R44] Fedorenko E. (2014). The role of domain-general cognitive control in language comprehension. Frontiers in Psychology, 5, 335. 24803909 10.3389/fpsyg.2014.00335 PMC4009428

[R45] Fedorenko E., Behr M. K., & Kanwisher N. (2011). Functional specificity for high-level linguistic processing in the human brain. Proceedings of the National Academy of Sciences, 108(39), 16428–16433. 10.1073/pnas.1112937108 PMC3182706 21885736

[R46] Fedorenko E., Blank I. A., Siegelman M., & Mineroff Z. (2020). Lack of selectivity for syntax relative to word meanings throughout the language network. Cognition, 203, 104348. 32569894 10.1016/j.cognition.2020.104348 PMC7483589

[R47] Fedorenko E., Duncan J., & Kanwisher N. (2013). Broad domain generality in focal regions of frontal and parietal cortex. Proceedings of the National Academy of Sciences, 110(41), 16616–16621. 10.1073/pnas.1315235110 PMC3799302 24062451

[R48] Fedorenko E., Hsieh P.-J., Nieto-Castañón A., Whitfield-Gabrieli S., & Kanwisher N. (2010). New method for fMRI investigations of language: Defining ROIs functionally in individual subjects. Journal of Neurophysiology, 104(2), 1177–1194. 20410363 10.1152/jn.00032.2010 PMC2934923

[R49] Fedorenko E., Ivanova A. A., & Regev T. I. (2024). The language network as a natural kind within the broader landscape of the human brain. 10.1038/s41583-024-00802-4 PMC13222024 38609551

[R50] Fedorenko E., Nieto-Castanon A., & Kanwisher N. (2012). Lexical and syntactic representations in the brain: An fMRI investigation with multi-voxel pattern analyses. Neuropsychologia, 50(4), 499–513. 21945850 10.1016/j.neuropsychologia.2011.09.014 PMC3292791

[R51] Fedorenko E., Scott T. L., Brunner P., Coon W. G., Pritchett B., Schalk G., & Kanwisher N. (2016). Neural correlate of the construction of sentence meaning. Proceedings of the National Academy of Sciences, 113(41), E6256–E6262. 10.1073/pnas.1612132113 PMC5068329 27671642

[R52] Fedorenko E., & Shain C. (2021). Similarity of computations across domains does not imply shared implementation: The case of language comprehension. Current Directions in Psychological Science, 30(6), 526–534. 35295820 10.1177/09637214211046955 PMC8923525

[R53] Ferreira F., & Bailey K. G. (2004). Disfluencies and human language comprehension. Trends in Cognitive Sciences, 8(5), 231–237. 15120682 10.1016/j.tics.2004.03.011

[R54] Ferreira F., Bailey K. G., & Ferraro V. (2002). Good-enough representations in language comprehension. Current Directions in Psychological Science, 11(1), 11–15.

[R55] Ferreira F., & Stacey J. (2000). The misinterpretation of passive sentences. Manuscript Submitted for Publication, 131.

[R56] Ferstl E. C., Neumann J., Bogler C., & Von Cramon D. Y. (2008). The extended language network: A meta-analysis of neuroimaging studies on text comprehension. Human Brain Mapping, 29(5), 581–593. 17557297 10.1002/hbm.20422 PMC2878642

[R57] Ferstl E. C., & Von Cramon D. Y. (2001). The role of coherence and cohesion in text comprehension: An event-related fMRI study. Cognitive Brain Research, 11(3), 325–340. 11339984 10.1016/s0926-6410(01)00007-6

[R58] Fiske A., & Holmboe K. (2019). Neural substrates of early executive function development. Developmental Review, 52, 42–62. 31417205 10.1016/j.dr.2019.100866 PMC6686207

[R59] Forster K. I., Guerrera C., & Elliot L. (2009). The maze task: Measuring forced incremental sentence processing time. Behavior Research Methods, 41, 163–171. 19182136 10.3758/BRM.41.1.163

[R60] Frank S. L., & Bod R. (2011). Insensitivity of the human sentence-processing system to hierarchical structure. Psychological Science, 22(6), 829–834. 21586764 10.1177/0956797611409589

[R61] Friederici A. D., Kotz S. A., Scott S. K., & Obleser J. (2010). Disentangling syntax and intelligibility in auditory language comprehension. Human Brain Mapping, 31(3), Article 3. 10.1002/hbm.20878 PMC6870868 19718654

[R62] Friston K. J., Ashburner J., Frith C. D., Poline J.-B., Heather J. D., & Frackowiak R. S. (1995). Spatial registration and normalization of images. Human Brain Mapping, 3(3), 165–189.

[R63] Frost M. A., & Goebel R. (2012). Measuring structural–functional correspondence: Spatial variability of specialised brain regions after macro-anatomical alignment. Neuroimage, 59(2), 1369–1381. 21875671 10.1016/j.neuroimage.2011.08.035

[R64] Gibson E. (2000). The dependency locality theory: A distance-based theory of linguistic complexity. In Image, language, brain: Papers from the first mind articulation project symposium (pp. 94–126). The MIT Press.

[R65] Gibson E., Bergen L., & Piantadosi S. T. (2013). Rational integration of noisy evidence and prior semantic expectations in sentence interpretation. Proceedings of the National Academy of Sciences, 110(20), 8051–8056. 10.1073/pnas.1216438110 PMC3657782 23637344

[R66] Gibson E., & Pearlmutter N. J. (1998). Constraints on sentence comprehension. Trends in Cognitive Sciences, 2(7), 262–268. 21244925 10.1016/S1364-6613(98)01187-5

[R67] Goldstein A., Zada Z., Buchnik E., Schain M., Price A., Aubrey B., Nastase S. A., Feder A., Emanuel D., & Cohen A. (2022). Shared computational principles for language processing in humans and deep language models. Nature Neuroscience, 25(3), 369–380. 35260860 10.1038/s41593-022-01026-4 PMC8904253

[R68] Goucha T., & Friederici A. D. (2015). The language skeleton after dissecting meaning: A functional segregation within Broca’s area. NeuroImage, 114, 294–302. 25871627 10.1016/j.neuroimage.2015.04.011

[R69] Grodner D., & Gibson E. (2005). Consequences of the serial nature of linguistic input for sentenial complexity. Cognitive Science, 29(2), 261–290. 21702774 10.1207/s15516709cog0000_7

[R70] Hagoort P., Brown C., & Groothusen J. (1993). The syntactic positive shift (SPS) as an ERP measure of syntactic processing. Language and Cognitive Processes, 8(4), 439–483.

[R71] Hagoort P., Hald L., Bastiaansen M., & Petersson K. M. (2004). Integration of word meaning and world knowledge in language comprehension. Science, 304(5669), 438–441. 15031438 10.1126/science.1095455

[R72] Heilbron M., Armeni K., Schoffelen J.-M., Hagoort P., & de Lange F. P. (2022). A hierarchy of linguistic predictions during natural language comprehension. Proceedings of the National Academy of Sciences, 119(32), Article 32. 10.1073/pnas.2201968119 PMC9371745 35921434

[R73] Hewitt J., & Manning C. D. (2019). A structural probe for finding syntax in word representations. Proceedings of the 2019 Conference of the North American Chapter of the Association for Computational Linguistics: Human Language Technologies, Volume 1 (Long and Short Papers), 4129–4138.

[R74] Hirsh-Pasek K., & Golinkoff R. M. (2010). Action meets word: How children learn verbs. Oxford University Press.

[R75] Holcomb P. J. (1993). Semantic priming and stimulus degradation: Implications for the role of the N400 in language processing. Psychophysiology, 30(1), 47–61. 8416062 10.1111/j.1469-8986.1993.tb03204.x

[R76] Honnibal M., Montani I., Van Landeghem S., Boyd A., & others. (2020). spaCy: Industrial-strength natural language processing in python.

[R77] Hu J., Small H., Kean H., Takahashi A., Zekelman L., Kleinman D., Ryan E., Nieto-Castañón A., Ferreira V., & Fedorenko E. (2023). Precision fMRI reveals that the language-selective network supports both phrase-structure building and lexical access during language production. Cerebral Cortex, 33(8), 4384–4404. 36130104 10.1093/cercor/bhac350 PMC10110436

[R78] Hugdahl K., Raichle M. E., Mitra A., & Specht K. (2015). On the existence of a generalized non-specific task-dependent network. Frontiers in Human Neuroscience, 9, 430. 26300757 10.3389/fnhum.2015.00430 PMC4526816

[R79] Humphries C., Binder J. R., Medler D. A., & Liebenthal E. (2006). Syntactic and semantic modulation of neural activity during auditory sentence comprehension. Journal of Cognitive Neuroscience, 18(4), 665–679. 16768368 10.1162/jocn.2006.18.4.665 PMC1635792

[R80] Humphries C., Love T., Swinney D., & Hickok G. (2005). Response of anterior temporal cortex to syntactic and prosodic manipulations during sentence processing. Human Brain Mapping, 26(2), 128–138. 15895428 10.1002/hbm.20148 PMC6871757

[R81] Ivanova A. A. (2022). The role of language in broader human cognition: Evidence from neuroscience [PhD Thesis]. Massachusetts Institute of Technology.

[R82] Ivanova A. A., Kauf C., Kanwisher N., Kean H., Goldhaber T., Mineroff Z., Balewski Z., Varley R., & Fedorenko E. (2022). Multiple brain regions show modality-invariant responses to event semantics. Society for the Neurobiology of Language.

[R83] Ivanova A. A., Mineroff Z., Zimmerer V., Kanwisher N., Varley R., & Fedorenko E. (2021). The language network is recruited but not required for nonverbal event semantics. Neurobiology of Language, 2(2), 176–201. 37216147 10.1162/nol_a_00030 PMC10158592

[R84] Ivanova A. A., Srikant S., Sueoka Y., Kean H. H., Dhamala R., O’Reilly U.-M., Bers M. U., & Fedorenko E. (2020). Comprehension of computer code relies primarily on domain-general executive brain regions. eLife, 9, e58906. 10.7554/eLife.58906 33319744 PMC7738192

[R85] Jackson R. L., Hoffman P., Pobric G., & Ralph M. A. L. (2016). The semantic network at work and rest: Differential connectivity of anterior temporal lobe subregions. Journal of Neuroscience, 36(5), 1490–1501. 26843633 10.1523/JNEUROSCI.2999-15.2016 PMC4737765

[R86] Jacoby N., & Fedorenko E. (2020). Discourse-level comprehension engages medial frontal Theory of Mind brain regions even for expository texts. Language, Cognition and Neuroscience, 35(6), 780–796. 32984430 10.1080/23273798.2018.1525494 PMC7518647

[R87] Joshi A. K. (1985). Tree adjoining grammars: How much context-sensitivity is required to provide reasonable structural descriptions?

[R88] Jouravlev O., Kell A. J., Mineroff Z., Haskins A. J., Ayyash D., Kanwisher N., & Fedorenko E. (2020). Reduced language lateralization in autism and the broader autism phenotype as assessed with robust individual-subjects analyses. Autism Research, 13(10), 1746–1761. 32935455 10.1002/aur.2393

[R89] Jouravlev O., Schwartz R., Ayyash D., Mineroff Z., Gibson E., & Fedorenko E. (2019). Tracking colisteners’ knowledge states during language comprehension. Psychological Science, 30(1), 3–19. 30444681 10.1177/0956797618807674 PMC6344950

[R90] Jouravlev O., Zheng D., Balewski Z., Pongos A. L. A., Levan Z., Goldin-Meadow S., & Fedorenko E. (2019). Speech-accompanying gestures are not processed by the language-processing mechanisms. Neuropsychologia, 132, 107132. 31276684 10.1016/j.neuropsychologia.2019.107132 PMC6708375

[R91] Julian J. B., Fedorenko E., Webster J., & Kanwisher N. (2012). An algorithmic method for functionally defining regions of interest in the ventral visual pathway. Neuroimage, 60(4), 2357–2364. 22398396 10.1016/j.neuroimage.2012.02.055

[R92] Kauf C., Ivanova A. A., Rambelli G., Chersoni E., She J. S., Chowdhury Z., Fedorenko E., & Lenci A. (2023). Event knowledge in large language models: The gap between the impossible and the unlikely. Cognitive Science, 47(11), e13386. 38009752 10.1111/cogs.13386

[R93] Keuleers E., & Brysbaert M. (2010). Wuggy: A multilingual pseudoword generator. Behavior Research Methods, 42, 627–633. 20805584 10.3758/BRM.42.3.627

[R94] Kim A., & Osterhout L. (2005). The independence of combinatory semantic processing: Evidence from event-related potentials. Journal of Memory and Language, 52(2), 205– 225.

[R95] Kuperberg G. R. (2016). Separate streams or probabilistic inference? What the N400 can tell us about the comprehension of events. Language, Cognition and Neuroscience, 31(5), 602– 616. 27570786 10.1080/23273798.2015.1130233 PMC4996121

[R96] Kuperberg G. R., Holcomb P. J., Sitnikova T., Greve D., Dale A. M., & Caplan D. (2003). Distinct patterns of neural modulation during the processing of conceptual and syntactic anomalies. Journal of Cognitive Neuroscience, 15(2), 272–293. 12676064 10.1162/089892903321208204

[R97] Kuperberg G. R., & Jaeger T. F. (2016). What do we mean by prediction in language comprehension? Language, Cognition and Neuroscience, 31(1), 32–59. 27135040 10.1080/23273798.2015.1102299 PMC4850025

[R98] Kuperberg G. R., Lakshmanan B. M., Caplan D. N., & Holcomb P. J. (2006). Making sense of discourse: An fMRI study of causal inferencing across sentences. Neuroimage, 33(1), 343–361. 16876436 10.1016/j.neuroimage.2006.06.001

[R99] Kutas M., & Hillyard S. A. (1984). Brain potentials during reading reflect word expectancy and semantic association. Nature, 307(5947), 161–163. 6690995 10.1038/307161a0

[R100] Lau E., Stroud C., Plesch S., & Phillips C. (2006). The role of structural prediction in rapid syntactic analysis. Brain and Language, 98(1), 74–88. 16620944 10.1016/j.bandl.2006.02.003

[R101] Leech G. N. (1992). 100 million words of English: The British National Corpus (BNC). Language Research.

[R102] Lerner Y., Honey C. J., Silbert L. J., & Hasson U. (2011). Topographic mapping of a hierarchy of temporal receptive windows using a narrated story. Journal of Neuroscience, 31(8), 2906–2915. 21414912 10.1523/JNEUROSCI.3684-10.2011 PMC3089381

[R103] Levy R. (2008a). A Noisy-Channel Model of Human Sentence Comprehension under Uncertain Input. In Lapata M. & Ng H. T. (Eds.), Proceedings of the 2008 Conference on Empirical Methods in Natural Language Processing (pp. 234–243). Association for Computational Linguistics. https://aclanthology.org/D08-1025

[R104] Levy R. (2008b). Expectation-based syntactic comprehension. Cognition, 106(3), 1126–1177. 17662975 10.1016/j.cognition.2007.05.006

[R105] Levy R. (2011). Integrating surprisal and uncertain-input models in online sentence comprehension: Formal techniques and empirical results. In Lin D., Matsumoto Y., & Mihalcea R. (Eds.), Proceedings of the 49th Annual Meeting of the Association for Computational Linguistics: Human Language Technologies (pp. 1055–1065). Association for Computational Linguistics. https://aclanthology.org/P11-1106

[R106] Levy R., Bicknell K., Slattery T., & Rayner K. (2009). Eye movement evidence that readers maintain and act on uncertainty about past linguistic input. Proceedings of the National Academy of Sciences, 106(50), 21086–21090. 10.1073/pnas.0907664106 PMC2795489 19965371

[R107] Lohse B., Hawkins J. A., & Wasow T. (2004). Domain minimization in English verb-particle constructions. Language, 238–261.

[R108] Loper E., & Bird S. (2002). Nltk: The natural language toolkit. arXiv Preprint Cs/0205028.

[R109] Lopopolo A., Frank S. L., van den Bosch A., & Willems R. M. (2017). Using stochastic language models (SLM) to map lexical, syntactic, and phonological information processing in the brain. PLoS ONE, 12(5), Article 5. 10.1371/journal.pone.0177794 PMC5436813 28542396

[R110] MacDonald M. C., Pearlmutter N. J., & Seidenberg M. S. (1994). The lexical nature of syntactic ambiguity resolution. Psychological Review, 101(4), 676. 7984711 10.1037/0033-295x.101.4.676

[R111] Mahowald K., Diachek E., Gibson E., Fedorenko E., & Futrell R. (2023). Grammatical cues to subjecthood are redundant in a majority of simple clauses across languages. Cognition, 241, 105543. 10.1016/j.cognition.2023.105543 37713956

[R112] Malik-Moraleda S., Ayyash D., Gallée J., Affourtit J., Hoffmann M., Mineroff Z., Jouravlev O., & Fedorenko E. (2022). An investigation across 45 languages and 12 language families reveals a universal language network. Nature Neuroscience, 25(8), 1014–1019. 35856094 10.1038/s41593-022-01114-5 PMC10414179

[R113] Marcus M., Santorini B., & Marcinkiewicz M. A. (1993). Building a Large Annotated Corpus of English: The Penn Treebank. Computational Linguistics, 19(2), 313–330.

[R114] Marslen-Wilson W., & Tyler L. K. (1975). Processing structure of sentence perception. Nature, 257(5529), 784–786. 1186856 10.1038/257784a0

[R115] Marslen-Wilson W., & Tyler L. K. (1980). The temporal structure of spoken language understanding. Cognition, 8(1), 1–71. 7363578 10.1016/0010-0277(80)90015-3

[R116] Matchin W., Hammerly C., & Lau E. (2017). The role of the IFG and pSTS in syntactic prediction: Evidence from a parametric study of hierarchical structure in fMRI. Cortex, 88, 106–123. 28088041 10.1016/j.cortex.2016.12.010

[R117] Matsuki K., Chow T., Hare M., Elman J. L., Scheepers C., & McRae K. (2011). Event-based plausibility immediately influences on-line language comprehension. Journal of Experimental Psychology: Learning, Memory, and Cognition, 37(4), 913. 21517222 10.1037/a0022964 PMC3130834

[R118] McRae K., & Matsuki K. (2009). People use their knowledge of common events to understand language, and do so as quickly as possible. Language and Linguistics Compass, 3(6), 1417–1429. 22125574 10.1111/j.1749-818X.2009.00174.x PMC3224485

[R119] Michel J.-B., Shen Y. K., Aiden A. P., Veres A., Gray M. K., Team G. B., Pickett J. P., Hoiberg D., Clancy D., & Norvig P. (2011). Quantitative analysis of culture using millions of digitized books. Science, 331(6014), 176–182. 21163965 10.1126/science.1199644 PMC3279742

[R120] Mikolov T., Sutskever I., Chen K., Corrado G. S., & Dean J. (2013). Distributed representations of words and phrases and their compositionality. Advances in Neural Information Processing Systems, 26.

[R121] Mineroff Z., Blank I. A., Mahowald K., & Fedorenko E. (2018). A robust dissociation among the language, multiple demand, and default mode networks: Evidence from inter-region correlations in effect size. Neuropsychologia, 119, 501–511. 10.1016/j.neuropsychologia.2018.09.011 30243926 PMC6191329

[R122] Mirault J., Snell J., & Grainger J. (2018). You that read wrong again! A transposed-word effect in grammaticality judgments. Psychological Science, 29(12), 1922–1929. 30355054 10.1177/0956797618806296

[R123] Mitchell D. J., Mousley A. L., Shafto M. A., Duncan J., & others. (2023). Neural contributions to reduced fluid intelligence across the adult lifespan. Journal of Neuroscience, 43(2), 293– 307. 36639907 10.1523/JNEUROSCI.0148-22.2022 PMC9838706

[R124] Mollica F., Siegelman M., Diachek E., Piantadosi S. T., Mineroff Z., Futrell R., Kean H., Qian P., & Fedorenko E. (2020). Composition is the core driver of the language-selective network. Neurobiology of Language, 1(1), 104–134. 36794007 10.1162/nol_a_00005 PMC9923699

[R125] Newman A. J., Pancheva R., Ozawa K., Neville H. J., & Ullman M. T. (2001). An event-related fMRI study of syntactic and semantic violations. Journal of Psycholinguistic Research, 30, 339–364. 11523278 10.1023/a:1010499119393

[R126] Nguyen L., Van Schijndel M., & Schuler W. (2012). Accurate unbounded dependency recovery using generalized categorial grammars. Proceedings of COLING 2012, 2125–2140.

[R127] Nieto-Castañón A. (2020). Handbook of functional connectivity magnetic resonance imaging methods in CONN. Hilbert Press.

[R128] Nieto-Castañón A., & Fedorenko E. (2012). Subject-specific functional localizers increase sensitivity and functional resolution of multi-subject analyses. NeuroImage, 63(3), Article 3. 10.1016/j.neuroimage.2012.06.065 PMC3477490 22784644

[R129] Nieuwland M. S., Martin A. E., & Carreiras M. (2013). Event-related brain potential evidence for animacy processing asymmetries during sentence comprehension. Brain and Language, 126(2), 151–158. 23735756 10.1016/j.bandl.2013.04.005

[R130] Nieuwland M. S., & Van Berkum J. J. (2006). When peanuts fall in love: N400 evidence for the power of discourse. Journal of Cognitive Neuroscience, 18(7), 1098–1111. 16839284 10.1162/jocn.2006.18.7.1098

[R131] Núñez-Peña M. I., & Honrubia-Serrano M. L. (2004). P600 related to rule violation in an arithmetic task. Cognitive Brain Research, 18(2), 130–141. 14736572 10.1016/j.cogbrainres.2003.09.010

[R132] Oldfield R. C. (1971). The assessment and analysis of handedness: The Edinburgh inventory. Neuropsychologia, 9(1), 97–113. 5146491 10.1016/0028-3932(71)90067-4

[R133] Osterhout L., & Holcomb P. J. (1992). Event-related brain potentials elicited by syntactic anomaly. Journal of Memory and Language, 31(6), 785–806.

[R134] Pallier C., Devauchelle A.-D., & Dehaene S. (2011). Cortical representation of the constituent structure of sentences. Proceedings of the National Academy of Sciences, 108(6), 2522– 2527. 10.1073/pnas.1018711108 PMC3038732 21224415

[R135] Patel A. D. (2003). Language, music, syntax and the brain. Nature Neuroscience, 6(7), 674–681. 12830158 10.1038/nn1082

[R136] Paunov A. M., Blank I. A., & Fedorenko E. (2019). Functionally distinct language and Theory of Mind networks are synchronized at rest and during language comprehension. Journal of Neurophysiology, 121(4), 1244–1265. 30601693 10.1152/jn.00619.2018 PMC6485726

[R137] Pennington J., Socher R., & Manning C. (2014). GloVe: Global Vectors for Word Representation. Proceedings of the 2014 Conference on Empirical Methods in Natural Language Processing (EMNLP), 1532–1543. 10.3115/v1/D14-1162

[R138] Pollard C., & Sag I. A. (1994). Head-Driven Phrase Structure Grammar. University of Chicago Press. https://press.uchicago.edu/ucp/books/book/chicago/H/bo3618318.html

[R139] Pritchett B. L., Hoeflin C., Koldewyn K., Dechter E., & Fedorenko E. (2018). High-level language processing regions are not engaged in action observation or imitation. Journal of Neurophysiology, 120(5), 2555–2570. 30156457 10.1152/jn.00222.2018 PMC6295536

[R140] Pullum G. K., & Gazdar G. (1982). Natural languages and context-free languages. Linguistics and Philosophy, 4, 471–504.

[R141] Radford A., Wu J., Child R., Luan D., Amodei D., & Sutskever I. (2019). Language models are unsupervised multitask learners. OpenAI Blog, 1(8), 9.

[R142] Regev T. I., Kim H. S., Chen X., Affourtit J., Schipper A. E., Bergen L., Mahowald K., & Fedorenko E. (2024). High-level language brain regions process sublexical regularities. Cerebral Cortex, 34(3), bhae077. 10.1093/cercor/bhae077 38494886 PMC11486690

[R143] Reuter-Lorenz P. A., Jonides J., Smith E. E., Hartley A., Miller A., Marshuetz C., & Koeppe R. A. (2000). Age differences in the frontal lateralization of verbal and spatial working memory revealed by PET. Journal of Cognitive Neuroscience, 12(1), 174–187. 10769314 10.1162/089892900561814

[R144] Roads B. D., & Love B. C. (2020). Learning as the unsupervised alignment of conceptual systems. Nature Machine Intelligence, 2(1), 76–82.

[R145] Ryskin R., Futrell R., Kiran S., & Gibson E. (2018). Comprehenders model the nature of noise in the environment. Cognition, 181, 141–150. 30195136 10.1016/j.cognition.2018.08.018 PMC6252256

[R146] Ryskin R., Stearns L., Bergen L., Eddy M., Fedorenko E., & Gibson E. (2021). An ERP index of real-time error correction within a noisy-channel framework of human communication. Neuropsychologia, 158, 107855. 33865848 10.1016/j.neuropsychologia.2021.107855

[R147] Sanford A. J., & Sturt P. (2002). Depth of processing in language comprehension: Not noticing the evidence. Trends in Cognitive Sciences, 6(9), 382–386. 12200180 10.1016/s1364-6613(02)01958-7

[R148] Saygın A. P., Wilson S. M., Dronkers N. F., & Bates E. (2004). Action comprehension in aphasia: Linguistic and non-linguistic deficits and their lesion correlates. Neuropsychologia, 42(13), 1788–1804. 15351628 10.1016/j.neuropsychologia.2004.04.016

[R149] Schettini E., Hiersche K. J., & Saygin Z. M. (2023). Individual variability in performance reflects selectivity of the multiple demand network among children and adults. Journal of Neuroscience, 43(11), 1940–1951. 36750368 10.1523/JNEUROSCI.1460-22.2023 PMC10027032

[R150] Scott T. L., Gallée J., & Fedorenko E. (2017). A new fun and robust version of an fMRI localizer for the frontotemporal language system. Cognitive Neuroscience, 8(3), 167–176. 27386919 10.1080/17588928.2016.1201466

[R151] Shain C., Blank I. A., Fedorenko E., Gibson E., & Schuler W. (2022). Robust Effects of Working Memory Demand during Naturalistic Language Comprehension in Language-Selective Cortex. Journal of Neuroscience, 42(39), Article 39. 10.1523/JNEUROSCI.1894-21.2022 PMC9525168 36002263

[R152] Shain C., Blank I. A., van Schijndel M., Schuler W., & Fedorenko E. (2020). fMRI reveals language-specific predictive coding during naturalistic sentence comprehension. Neuropsychologia, 138, 107307. 10.1016/j.neuropsychologia.2019.107307 31874149 PMC7140726

[R153] Shain C., Kean H., Casto C., Lipkin B., Affourtit J., Siegelman M., Mollica F., & Fedorenko E. (in press). Graded sensitivity to structure and meaning throughout the human language network. Journal of Cognitive Neuroscience. 10.1162/jocn_a_02164 38683732

[R154] Shain C., Meister C., Pimentel T., Cotterell R., & Levy R. (2024). Large-scale evidence for logarithmic effects of word predictability on reading time. Proceedings of the National Academy of Sciences. 10.1073/pnas.2307876121 PMC10927576 38422017

[R155] Shain C., Paunov A., Chen X., Lipkin B., & Fedorenko E. (2023). No evidence of theory of mind reasoning in the human language network. Cerebral Cortex, 33(10), 6299–6319. 36585774 10.1093/cercor/bhac505 PMC10183748

[R156] Shashidhara S., Mitchell D. J., Erez Y., & Duncan J. (2019). Progressive recruitment of the frontoparietal multiple-demand system with increased task complexity, time pressure, and reward. Journal of Cognitive Neuroscience, 31(11), 1617–1630. 31274390 10.1162/jocn_a_01440 PMC7116493

[R157] Sinha K., Parthasarathi P., Pineau J., & Williams A. (2021). UnNatural Language Inference. Proceedings of the 59th Annual Meeting of the Association for Computational Linguistics and the 11th International Joint Conference on Natural Language Processing (Volume 1: Long Papers), 7329–7346.

[R158] Smith N. J. (2014). ZS: A file format for efficiently distributing, using, and archiving record-oriented data sets of any size. Manuscript Submitted for Publication. School of Informatics, University of Edinburgh. Retrieved from Http://Vorpus.Org/Papers/Draft/Zs-Paper.Pdf.

[R159] Smith N. J., & Levy R. (2013). The effect of word predictability on reading time is logarithmic. Cognition, 128(3), Article 3. 10.1016/j.cognition.2013.02.013 PMC3709001 23747651

[R160] Spelke E. S. (2023). Core knowledge, language learning, and the origins of morality and pedagogy: Reply to reviews of What babies know. Mind & Language, 38(5), 1336–1350.

[R161] Spivey M. J., & Tanenhaus M. K. (1998). Syntactic ambiguity resolution in discourse: Modeling the effects of referential context and lexical frequency. Journal of Experimental Psychology: Learning, Memory, and Cognition, 24(6), 1521. 9835064 10.1037//0278-7393.24.6.1521

[R162] Steedman M. (2000). The Syntactic Process. A Bradford Book.

[R163] Sullivan G. M., & Feinn R. (2012). Using effect size—Or why the P value is not enough. Journal of Graduate Medical Education, 4(3), 279–282. 23997866 10.4300/JGME-D-12-00156.1 PMC3444174

[R164] Swets B., Desmet T., Clifton C., & Ferreira F. (2008). Underspecification of syntactic ambiguities: Evidence from self-paced reading. Memory & Cognition, 36, 201–216. 18323075 10.3758/mc.36.1.201

[R165] Tabor W., & Hutchins S. (2004). Evidence for self-organized sentence processing: Digging-in effects. Journal of Experimental Psychology: Learning, Memory, and Cognition, 30(2), 431. 14979816 10.1037/0278-7393.30.2.431

[R166] Tahmasebi A. M., Davis M. H., Wild C. J., Rodd J. M., Hakyemez H., Abolmaesumi P., & Johnsrude I. S. (2012). Is the link between anatomical structure and function equally strong at all cognitive levels of processing? Cerebral Cortex, 22(7), 1593–1603. 21893681 10.1093/cercor/bhr205

[R167] Tanenhaus M. K., Spivey-Knowlton M. J., Eberhard K. M., & Sedivy J. C. (1995). Integration of visual and linguistic information in spoken language comprehension. Science, 268(5217), 1632–1634. 7777863 10.1126/science.7777863

[R168] Tucker M., Qian P., & Levy R. (2021). What if This Modified That? Syntactic Interventions with Counterfactual Embeddings. Findings of the Association for Computational Linguistics: ACL-IJCNLP 2021, 862–875. 10.18653/v1/2021.findings-acl.76

[R169] Van Schijndel M., & Schuler W. (2013). An analysis of frequency-and memory-based processing costs. Proceedings of the 2013 Conference of the North American Chapter of the Association for Computational Linguistics: Human Language Technologies, 95–105.

[R170] Varley R., & Siegal M. (2000). Evidence for cognition without grammar from causal reasoning and ‘theory of mind’in an agrammatic aphasic patient. Current Biology, 10(12), 723–726. 10873809 10.1016/s0960-9822(00)00538-8

[R171] Wang L., Brothers T., Jensen O., & Kuperberg G. R. (2023). Dissociating the pre-activation of word meaning and form during sentence comprehension: Evidence from EEG representational similarity analysis. Psychonomic Bulletin & Review, 1–12. 10.3758/s13423-023-02385-0 PMC10985416 37783897

[R172] Warren T., & Dickey M. W. (2021). The use of linguistic and world knowledge in language processing. Language and Linguistics Compass, 15(4), e12411.

[R173] Wen Y., Mirault J., & Grainger J. (2021). The transposed-word effect revisited: The role of syntax in word position coding. Language, Cognition and Neuroscience, 36(5), 668–673. 35391898 10.1080/23273798.2021.1880608 PMC8979551

[R174] Wirth M., Jann K., Dierks T., Federspiel A., Wiest R., & Horn H. (2011). Semantic memory involvement in the default mode network: A functional neuroimaging study using independent component analysis. Neuroimage, 54(4), 3057–3066. 20965253 10.1016/j.neuroimage.2010.10.039

[R175] Wolf T., Debut L., Sanh V., Chaumond J., Delangue C., Moi A., Cistac P., Rault T., Louf R., Funtowicz M., & others. (2020). Transformers: State-of-the-art natural language processing. Proceedings of the 2020 Conference on Empirical Methods in Natural Language Processing: System Demonstrations, 38–45.

[R176] Wu W., & Hoffman P. (2023). Age differences in the neural processing of semantics, within and beyond the core semantic network. Neurobiology of Aging, 131, 88–105. 37603932 10.1016/j.neurobiolaging.2023.07.022

[R177] Wurm M. F., & Caramazza A. (2019). Distinct roles of temporal and frontoparietal cortex in representing actions across vision and language. Nature Communications, 10(1), 289. 10.1038/s41467-018-08084-y PMC6336825 30655531

[R178] Zhang Y., Kauf C., Levy R. P., & Gibson E. (2024). Comparative illusions are evidence of rational inference in language comprehension. 10.1037/xge0001807 40689922

