# Supplementary material for: Linguistic inputs must be syntactically parsable to fully engage the language network
Source: bioRxiv. 2024 Jun 21:2024.06.21.599332. Preprint. [Version 1] doi: 10.1101/2024.06.21.599332 (PMC11212959; doi:10.1101/2024.06.21.599332)
Supplement: Supplement 1 [file NIHPP2024.06.21.599332v1-supplement-1.pdf]

bioRxiv preprint doi: https://doi.org/10.1101/2024.06.21.599332; this version posted June 22, 2024. The copyright holder for this preprint (which was not certified by peer review) is the author/funder, who has granted bioRxiv a license to display the preprint in perpetuity. It is made available under aCC-BY 4.0 International license.

Supplementary Information

Supplementary Figures

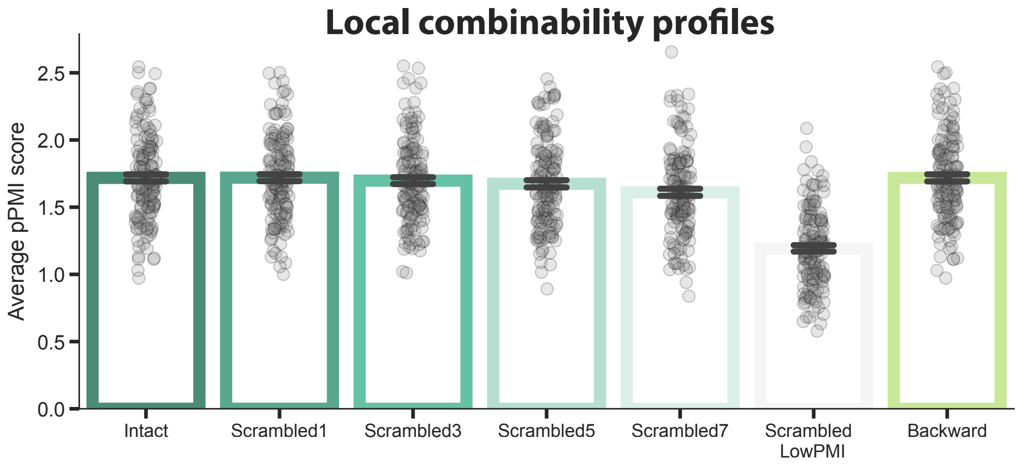

Figure SI 1. PMI calculation for the full set of materials used in Mollica, Siegelman et al.'s (2020) experiment 1, and the full set of our stimuli from the *Backward* condition.

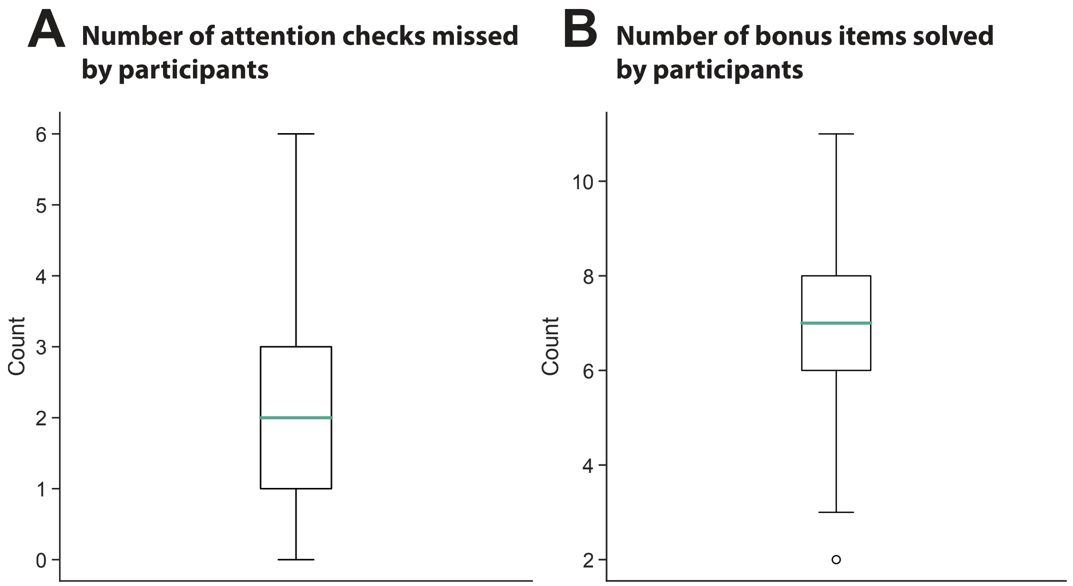

Figure SI 2. Participant exclusion criteria used for the behavioral reconstruction experiment. We used the error distributions for attention check and bonus items to determine the thresholds for exclusion. We excluded the lowest participants in the highest quartile for the number of attention checks missed, and in the lowest quartile for the number of bonus items solved.

bioRxiv preprint doi: https://doi.org/10.1101/2024.06.21.599332; this version posted June 22, 2024. The copyright holder for this preprint (which was not certified by peer review) is the author/funder, who has granted bioRxiv a license to display the preprint in perpetuity. It is made available under aCC-BY 4.0 International license.

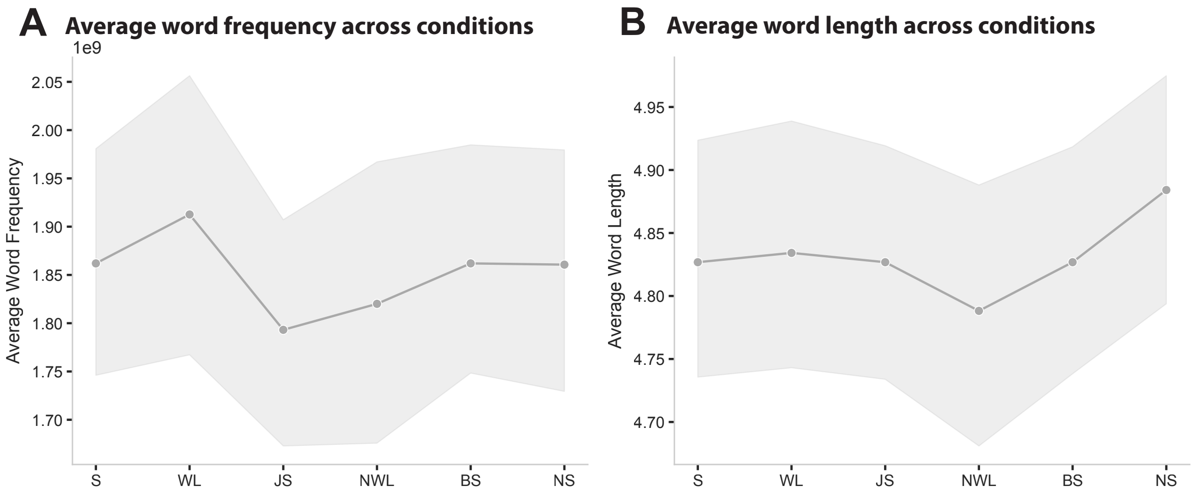

Figure SI 3. Low level controls for our experimental conditions. Stimuli are roughly matched in average word frequency and in average word length. Frequency was operationalized as the log of the number of occurrences of the word/phrase in the 2012 Google NGram corpus. Laplace smoothing was applied prior to taking the log. Word length indicates the average number of characters per word across the stimuli in a given condition.

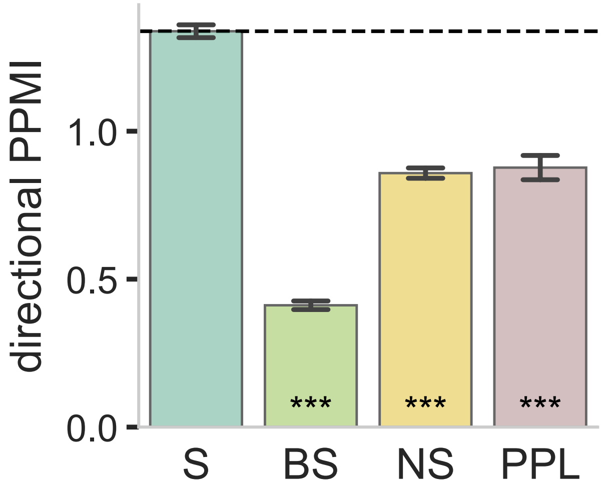

Figure SI 4. Hypothesis profile derived from directional PPMI measure. Predictions derived from a variation of the PPMI model described in 2.2.1 Critical task design and materials that considers word order, i.e., calculates the co-occurrence of the *ordered* bigram $w_i w_j$. Significant difference to the *Sentence* condition was established via post hoc pairwise t-tests, with p-values corrected for multiple comparisons using the Bonferroni procedure. The ordered PPMI measure underpredicts the language network activity in response to *Nonsense* stimuli (and overpredicts the response to *Predictable Phrase Lists* (**Figure SI 13**)).

bioRxiv preprint doi: https://doi.org/10.1101/2024.06.21.599332; this version posted June 22, 2024. The copyright holder for this preprint (which was not certified by peer review) is the author/funder, who has granted bioRxiv a license to display the preprint in perpetuity. It is made available under aCC-BY 4.0 International license.

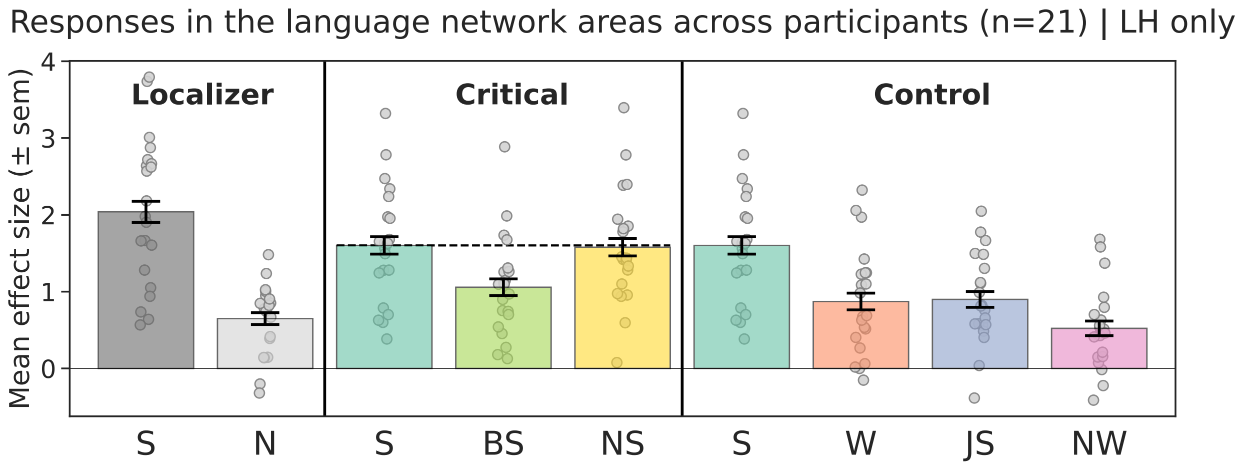

Figure SI 5. fMRI results for left-hemisphere language regions only. Neural responses (in % BOLD signal change relative to fixation) to the conditions of the language localizer and critical and control experimental conditions within the language network (averaged across all five fROIs) when including the LH (instead of the RH) language fROIs for the right-lateralized participants.

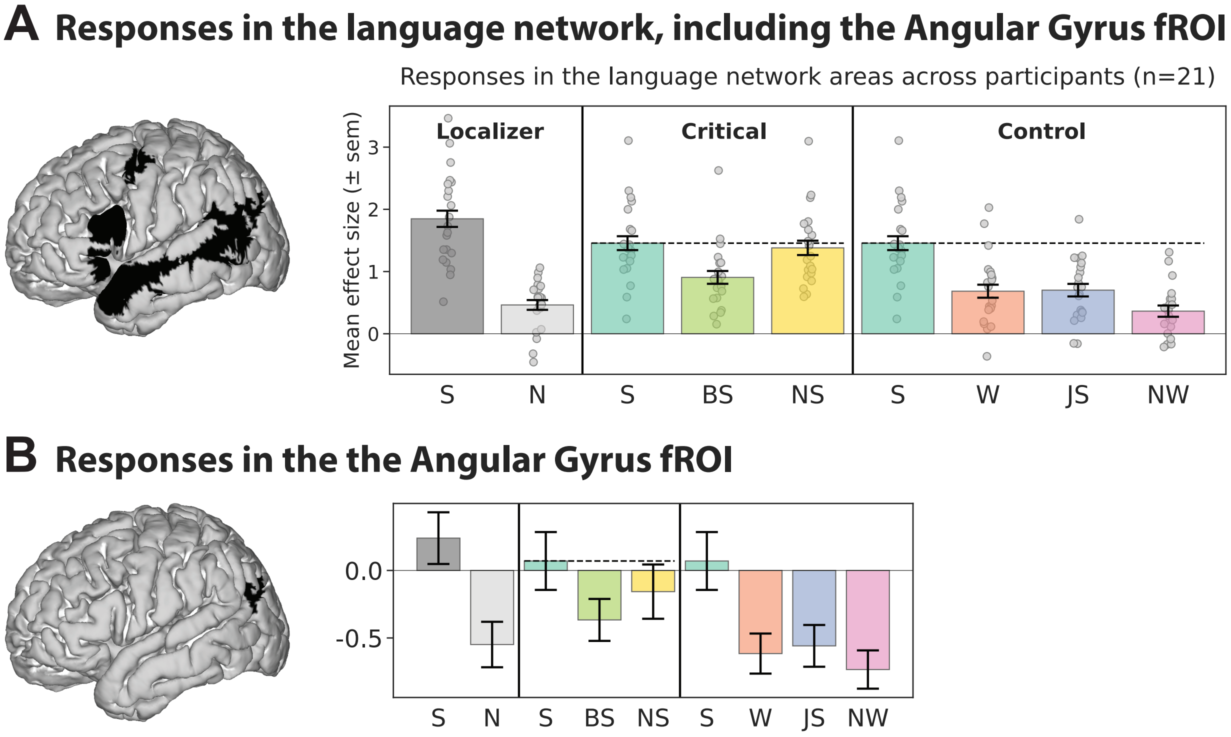

Figure SI 6. Responses in the language network, including the Angular Gyrus fROI. A) Neural responses (in % BOLD signal change relative to fixation) to the conditions of the language localizer and critical and control experimental conditions within the language network when including the Angular Gyrus fROI. **B)** Responses in just the Angular Gyrus fROI.

bioRxiv preprint doi: https://doi.org/10.1101/2024.06.21.599332; this version posted June 22, 2024. The copyright holder for this preprint (which was not certified by peer review) is the author/funder, who has granted bioRxiv a license to display the preprint in perpetuity. It is made available under aCC-BY 4.0 International license.

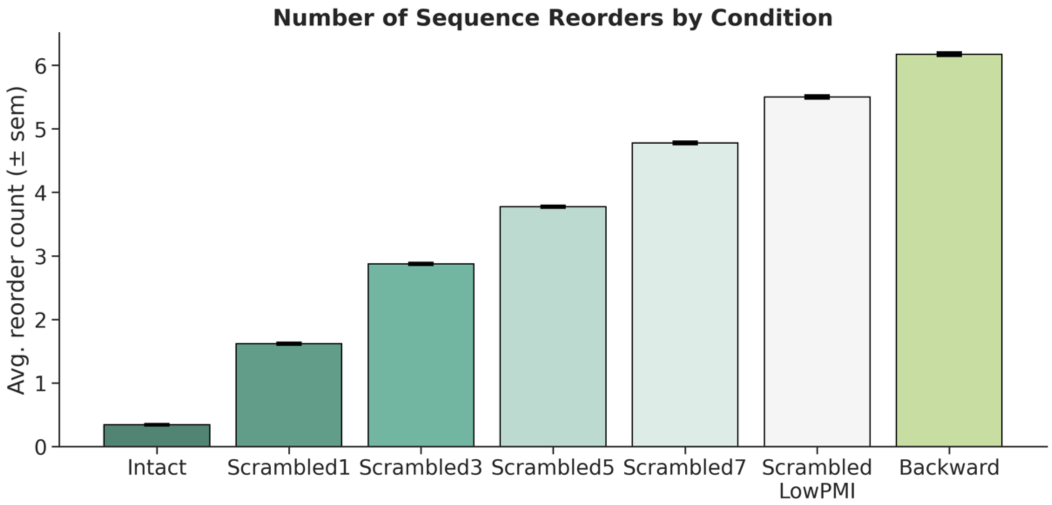

Figure SI 7. Validation of SynReco behavioral reconstruction paradigm. Participants actively reorder words during incremental sentence processing: each increase in the number of local word swaps led to an incremental increase in the number of time steps at which participants reordered the available words on the screen.

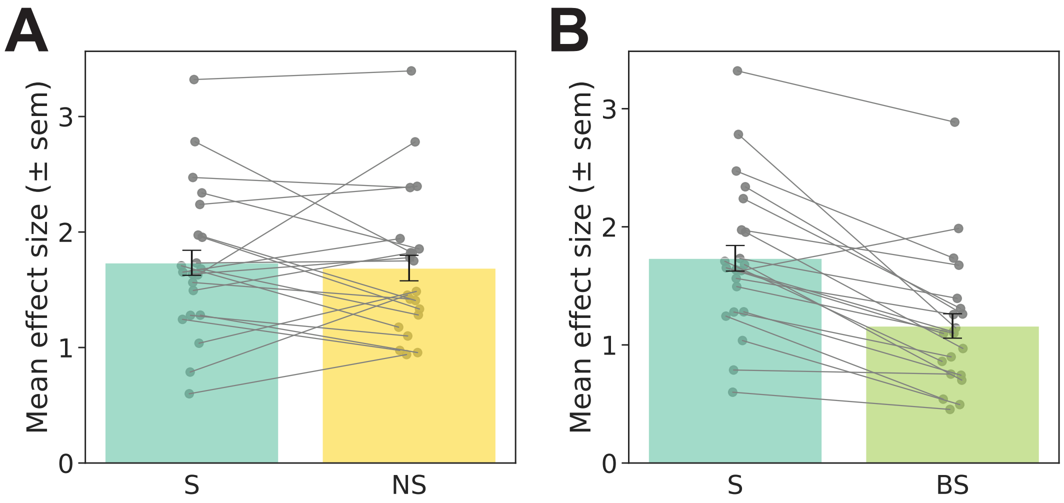

Figure SI 8. Individual subject effects for the critical conditions. Even though there is individual variability, the trends observed at the population level mostly hold in individual subjects, as well.

bioRxiv preprint doi: https://doi.org/10.1101/2024.06.21.599332; this version posted June 22, 2024. The copyright holder for this preprint (which was not certified by peer review) is the author/funder, who has granted bioRxiv a license to display the preprint in perpetuity. It is made available under aCC-BY 4.0 International license.

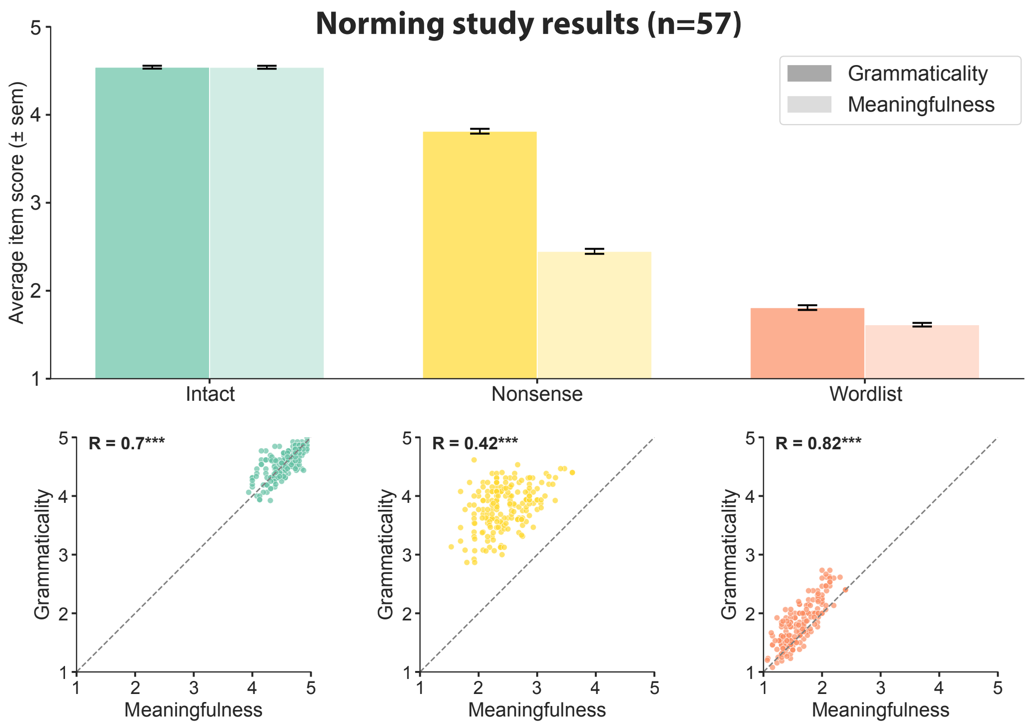

Figure SI 9. Full set of results from the behavioral norming study (Figure 4A). We asked participants to rate our *Plausible Sentence*, *Nonsense Sentence*, and *Word List* stimuli for two features: grammaticality and meaningfulness. *Nonsense Sentence* stimuli successfully dissociate the two features, even though they tend to be correlated. Nevertheless, *Nonsense Sentence* stimuli were rated worse grammatically than *Plausible Sentence* stimuli.

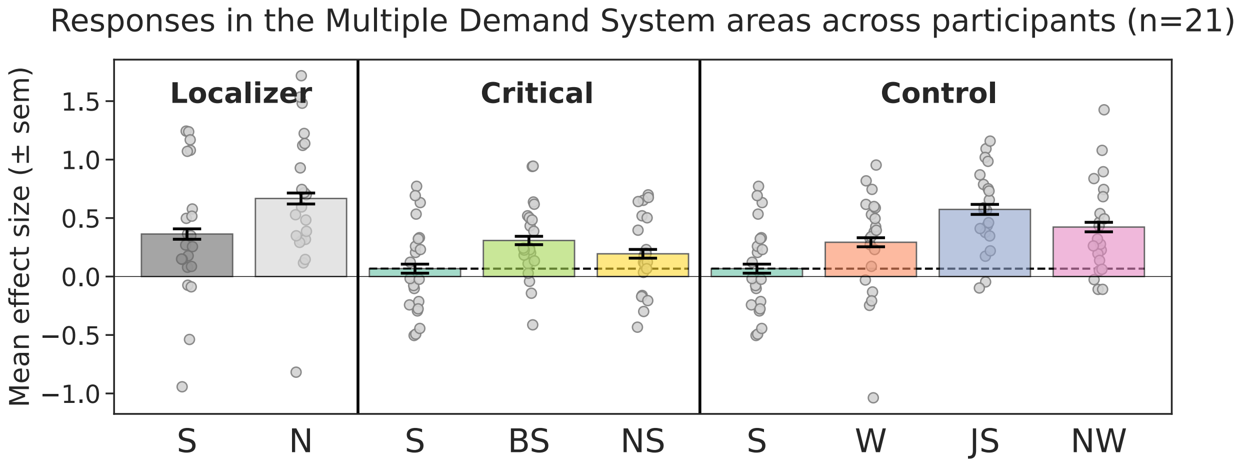

Figure SI 10. Multiple Demand system response. Neural responses (in % BOLD signal change relative to fixation) to the conditions of the language localizer and experimental conditions in the Multiple Demand (MD) system.

bioRxiv preprint doi: https://doi.org/10.1101/2024.06.21.599332; this version posted June 22, 2024. The copyright holder for this preprint (which was not certified by peer review) is the author/funder, who has granted bioRxiv a license to display the preprint in perpetuity. It is made available under aCC-BY 4.0 International license.

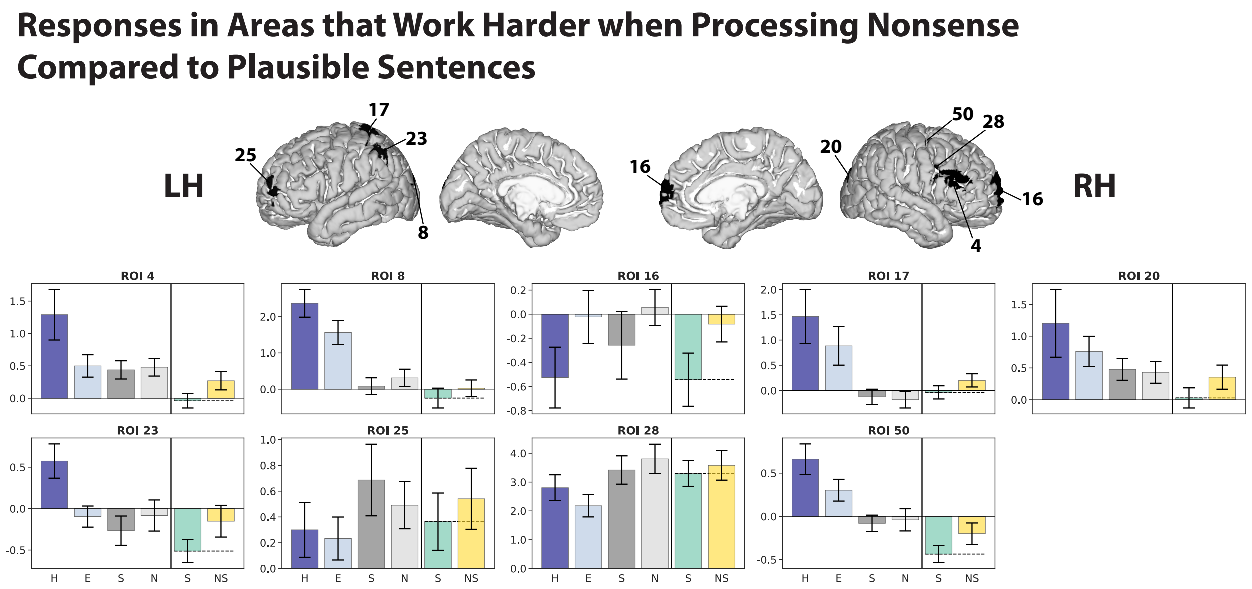

Figure SI 11. Finding regions that work harder when processing *Nonsense* sentences relative to *Plausible* sentences. The GSS whole-brain analysis for the *Nonsense sentence* > *Plausible sentence* contrast recovers a large network of brain regions. Follow-up analyses looking at the replicability of the contrast effect when including all subjects finds a subset of n=9 fROIs that show significant effects (shown here; average response shown in **Figure 4D**). We show the corresponding neural responses (in % BOLD signal change relative to fixation) within these fROIs to the conditions of the Multiple Demand (MD), language localizer and experimental conditions. However, these regions do not survive corrections for multiple comparisons across the entire network.

bioRxiv preprint doi: https://doi.org/10.1101/2024.06.21.599332; this version posted June 22, 2024. The copyright holder for this preprint (which was not certified by peer review) is the author/funder, who has granted bioRxiv a license to display the preprint in perpetuity. It is made available under aCC-BY 4.0 International license.

Responses in Areas that Work Harder when Processing Plausible Compared to Nonsense Sentences

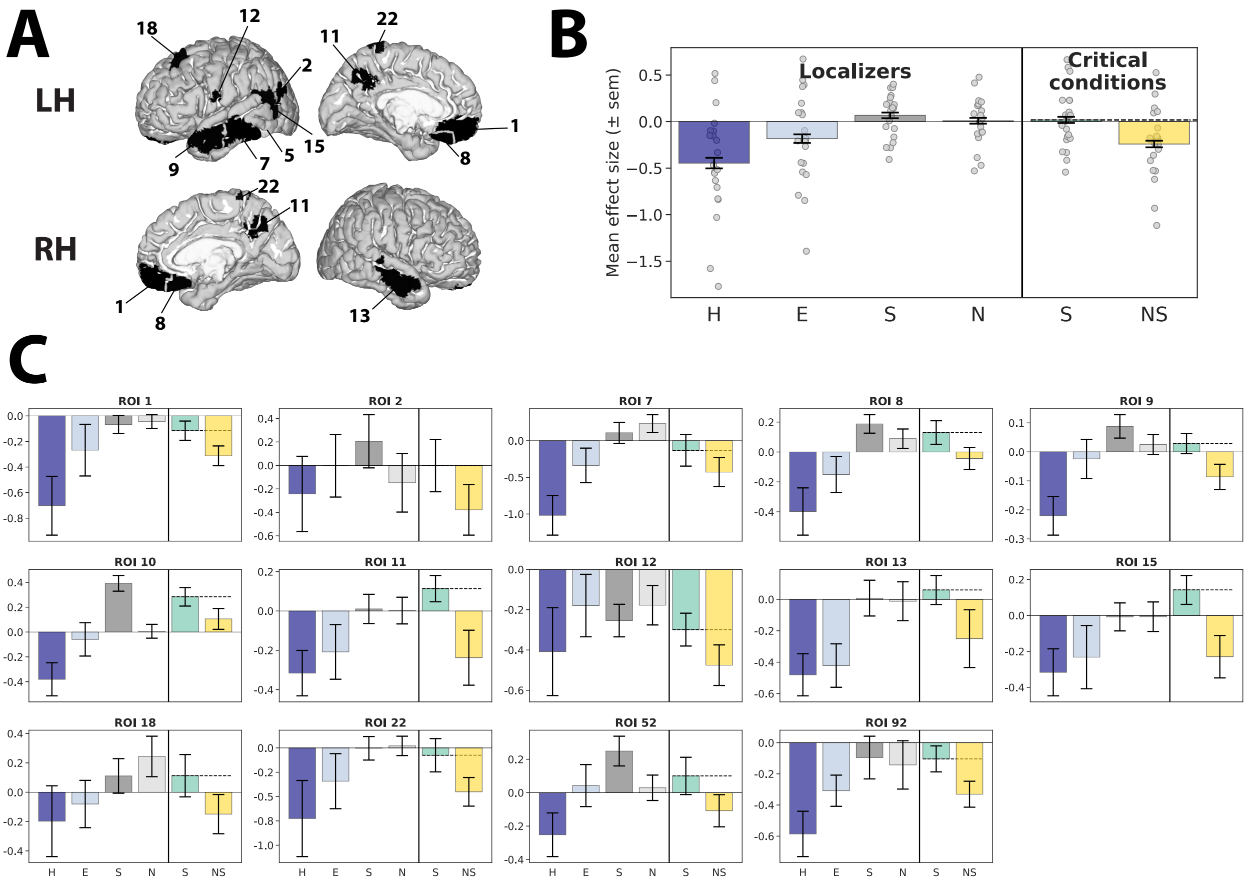

Figure SI 12. Finding regions that work harder when processing *Plausible* sentences relative to *Nonsense* sentences. A) The fROIs identified through GSS whole-brain analysis for the *Plausible sentence* > *Nonsense sentence* contrast, in which follow-up analyses looking at the replicability of the contrast effect when including all subjects show significant effects. However, only some of these regions survive corrections for multiple comparisons across the entire network. **B)** Neural responses (in % BOLD signal change relative to fixation) averaged across these fROIs to the conditions of the Multiple Demand (MD), language localizer and experimental conditions. **C)** Individual fROI responses.

bioRxiv preprint doi: https://doi.org/10.1101/2024.06.21.599332; this version posted June 22, 2024. The copyright holder for this preprint (which was not certified by peer review) is the author/funder, who has granted bioRxiv a license to display the preprint in perpetuity. It is made available under aCC-BY 4.0 International license.

A Sample Stimulus

Predictable Phrase List (PPL)	the herring the goldfish the flounder the pollock the cod
Unpredictable Phrase List (UPL)	the tiger the painter the genie the narcissus the brie the motel

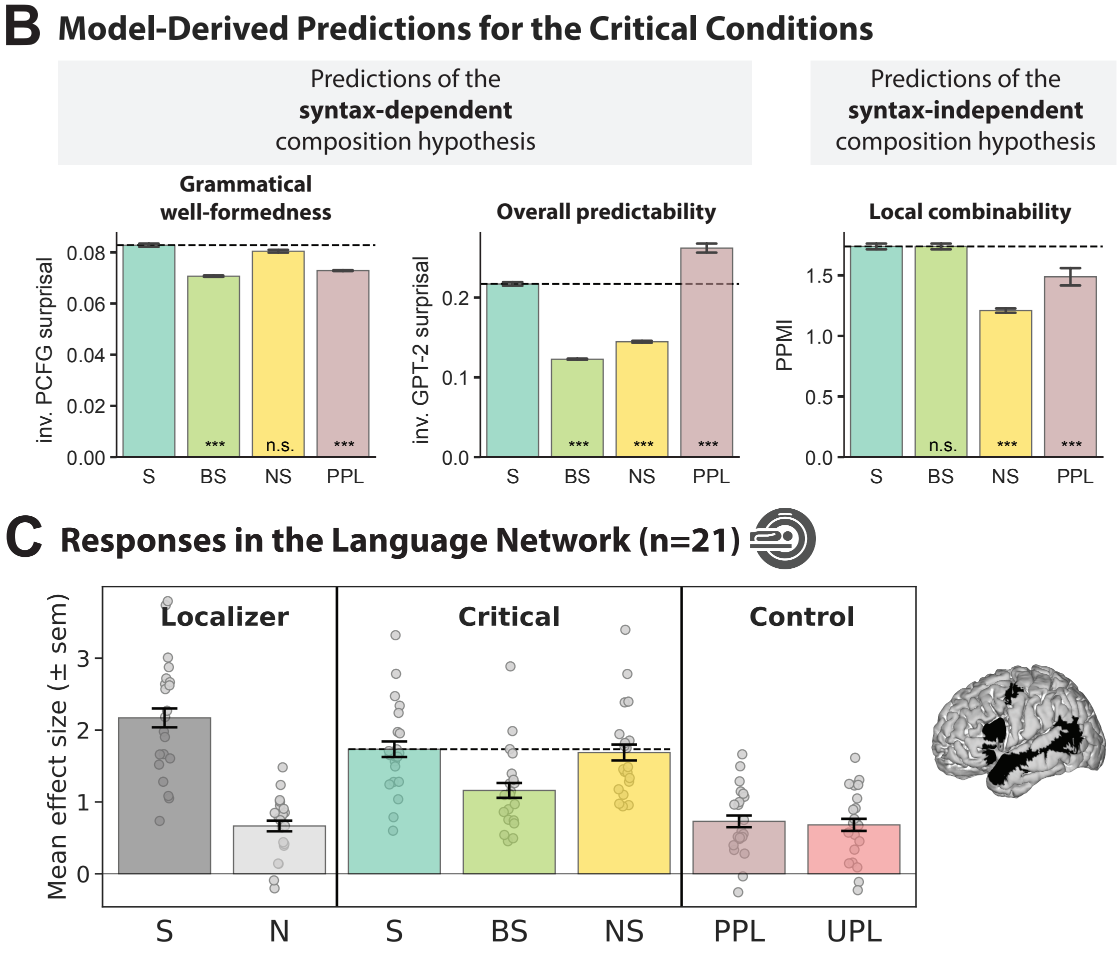

Figure SI 13. Language network response to semantically predictable and semantically unpredictable phrase lists. A) A sample item for the predictable and unpredictable phrase list conditions. Both conditions are made up of concatenated determiner phrases of the form `*the noun'*; for the predictable phrase list condition (PPL), nouns are drawn from a semantically coherent category (here: kinds of fish), for control condition, unpredictable phrase lists (UPL), nouns are randomly drawn from the categories used to design the PPL stimuli. **B)** Quantitatively derived predictions for syntax-dependent vs. syntax-independent semantic composition hypotheses. The syntax-dependent panel is split up into predictions derived via structure-mediated vs. expectation-mediated incremental processing models (see Discussion). To match the expected direction of the neural responses in the language network, we show inverse surprisal (i.e., the reciprocal of surprisal) for the PCFG and GPT-2 models. Significant difference to the *Sentence* condition was established via post hoc pairwise t-tests, with p-values corrected for multiple comparisons using the Bonferroni procedure. **D)** Neural responses (in % BOLD signal change relative to fixation) to the conditions of the language localizer and critical and control experimental conditions within the language network (averaged across the five regions, see brain

bioRxiv preprint doi: https://doi.org/10.1101/2024.06.21.599332; this version posted June 22, 2024. The copyright holder for this preprint (which was not certified by peer review) is the author/funder, who has granted bioRxiv a license to display the preprint in perpetuity. It is made available under aCC-BY 4.0 International license.

on the right). Dots show individual subject responses; error bars show standard errors of the mean by participants. The observed response shows that, in the absence of complex meaning, having a highly predictable stimulus (see panel B, Overall predictability) is insufficient to engage the language network.

bioRxiv preprint doi: https://doi.org/10.1101/2024.06.21.599332; this version posted June 22, 2024. The copyright holder for this preprint (which was not certified by peer review) is the author/funder, who has granted bioRxiv a license to display the preprint in perpetuity. It is made available under aCC-BY 4.0 International license.

Supplementary Tables

	Comparison with *Intact*		
	T-test statistic	Adjusted p-value	Cohen's d
Scr1	0.316	1.000	0.014
Scr3	1.840	0.522	0.121
Scr5	3.953**	0.003	0.303
Scr7	4.737***	0.000	0.575
ScrLowPMI	12.875***	0.000	2.207
Backward	0.201	1.000	0.000

Table SI 1. Statistics for PPMI stimuli characterization (Results; Section 3.1). Pairwise, two-sided, dependent t-tests for all comparisons performed between the PPMI values for the *Intact* and all conditions of interest. P-values were corrected for multiple comparisons using the Bonferroni procedure. Effect sizes, as quantified by Cohen's d are reported.

	Estimate	Est. error	95% CI	
Plausible Sentence (Sentence)	1.46*	0.47	0.50	2.38
Backward sentence vs. Sentence	−0.55*	0.13	−0.79	−0.30
Nonsense sentence vs. Sentence	−0.07	0.15	−0.35	0.21
Word list vs. Sentence	−0.77*	0.12	−1.01	−0.52
Jabberwocky sentence vs. Sentence	−0.76*	0.13	−1.01	−0.50
Nonword list vs. Sentence	−1.10*	0.14	−1.37	−0.83

Table SI 2. Results of mixed-effects linear regression for fMRI responses within the language network when including the Angular Gyrus fROI. Stimulus type was dummy-coded with *Sentence* as the reference level. *Denotes significant difference.

bioRxiv preprint doi: https://doi.org/10.1101/2024.06.21.599332; this version posted June 22, 2024. The copyright holder for this preprint (which was not certified by peer review) is the author/funder, who has granted bioRxiv a license to display the preprint in perpetuity. It is made available under aCC-BY 4.0 International license.

	group1	group2	meandiff	p-adj	lower	upper	reject
Experimental Items	Backward	Intact	-1.812	0.000	-2.542	-1.083	TRUE
	Backward	Scrambled1	-1.234	0.000	-1.964	-0.505	TRUE
	Backward	Scrambled3	-0.459	0.502	-1.188	0.270	FALSE
	Backward	Scrambled5	-0.416	0.618	-1.145	0.313	FALSE
	Backward	Scrambled7	-0.198	0.984	-0.927	0.531	FALSE
	Backward	Scrambled_LowPMI	-0.182	0.990	-0.911	0.547	FALSE
	Intact	Scrambled1	0.578	0.222	-0.151	1.307	FALSE
	Intact	Scrambled3	1.354	0.000	0.625	2.083	TRUE
	Intact	Scrambled5	1.396	0.000	0.667	2.125	TRUE
	Intact	Scrambled7	1.615	0.000	0.886	2.344	TRUE
	Intact	Scrambled_LowPMI	1.631	0.000	0.902	2.360	TRUE
	Scrambled1	Scrambled3	0.776	0.029	0.047	1.505	TRUE
	Scrambled1	Scrambled5	0.818	0.017	0.089	1.547	TRUE
	Scrambled1	Scrambled7	1.037	0.001	0.308	1.766	TRUE
	Scrambled1	Scrambled_LowPMI	1.053	0.001	0.324	1.782	TRUE
	Scrambled3	Scrambled5	0.043	1.000	-0.687	0.772	FALSE
	Scrambled3	Scrambled7	0.261	0.938	-0.468	0.990	FALSE
	Scrambled3	Scrambled_LowPMI	0.277	0.918	-0.452	1.006	FALSE
	Scrambled5	Scrambled7	0.219	0.974	-0.511	0.948	FALSE
	Scrambled5	Scrambled_LowPMI	0.235	0.963	-0.495	0.964	FALSE

bioRxiv preprint doi: https://doi.org/10.1101/2024.06.21.599332; this version posted June 22, 2024. The copyright holder for this preprint (which was not certified by peer review) is the author/funder, who has granted bioRxiv a license to display the preprint in perpetuity. It is made available under aCC-BY 4.0 International license.

	Scrambled7	Scrambled_ LowPMI	0.016	1.000	-0.713	0.745	FALSE
	group1	group2	meandiff	p-adj	lower	upper	reject
Reconstructed Items	Backward	Intact	-0.632	0.000	-0.858	-0.406	TRUE
	Backward	Scrambled1	-0.573	0.000	-0.799	-0.347	TRUE
	Backward	Scrambled3	-0.482	0.000	-0.708	-0.256	TRUE
	Backward	Scrambled5	-0.381	0.000	-0.607	-0.155	TRUE
	Backward	Scrambled7	-0.373	0.000	-0.599	-0.147	TRUE
	Backward	Scrambled_ LowPMI	-0.017	1.000	-0.243	0.209	FALSE
	Intact	Scrambled1	0.059	0.987	-0.167	0.285	FALSE
	Intact	Scrambled3	0.150	0.440	-0.076	0.376	FALSE
	Intact	Scrambled5	0.251	0.018	0.025	0.477	TRUE
	Intact	Scrambled7	0.259	0.013	0.033	0.485	TRUE
	Intact	Scrambled_ LowPMI	0.615	0.000	0.389	0.841	TRUE
	Scrambled1	Scrambled3	0.091	0.900	-0.135	0.317	FALSE
	Scrambled1	Scrambled5	0.192	0.157	-0.034	0.418	FALSE
	Scrambled1	Scrambled7	0.199	0.125	-0.027	0.425	FALSE
	Scrambled1	Scrambled_ LowPMI	0.555	0.000	0.330	0.781	TRUE
	Scrambled3	Scrambled5	0.101	0.843	-0.125	0.327	FALSE
	Scrambled3	Scrambled7	0.109	0.793	-0.118	0.335	FALSE
	Scrambled3	Scrambled_ LowPMI	0.465	0.000	0.239	0.691	TRUE
	Scrambled5	Scrambled7	0.007	1.000	-0.219	0.233	FALSE

bioRxiv preprint doi: https://doi.org/10.1101/2024.06.21.599332; this version posted June 22, 2024. The copyright holder for this preprint (which was not certified by peer review) is the author/funder, who has granted bioRxiv a license to display the preprint in perpetuity. It is made available under aCC-BY 4.0 International license.

	Scrambled5	Scrambled_LowPMI	0.363	0.000	0.138	0.589	TRUE
	Scrambled7	Scrambled_LowPMI	0.356	0.000	0.130	0.582	TRUE

Table SI 3. Statistics for Figure 1C in the main text (Results; Section 3.1). Multiple comparisons of group means using Tukey's Honestly Significant Difference (HSD) test.

	Estimate	Est. Error	95% CI	
Grand mean	-0.74*	0.13	-1.00	-0.47
Scr1 - Int	-2.02*	0.45	-2.96	-1.14
Scr3 - Scr1	-1.23*	0.41	-2.04	-0.43
Scr5 - Scr3	-0.35*	0.41	-1.15	0.44
Scr7 - Scr5	-1.07*	0.42	-1.90	-0.23
ScrLowPMI - Scr7	-1.46*	0.46	-2.38	-0.57
Backward - ScrLowPMI	-0.20	0.50	-1.19	0.77

Table SI 4. Statistics for verbatim reconstruction (Results; Section 3.1; Figure 1E). The results of a mixed-effect logistic regression model with a fixed effect and random slopes for Condition, and random effects for Participant and Item. *Denotes significant difference.

bioRxiv preprint doi: https://doi.org/10.1101/2024.06.21.599332; this version posted June 22, 2024. The copyright holder for this preprint (which was not certified by peer review) is the author/funder, who has granted bioRxiv a license to display the preprint in perpetuity. It is made available under aCC-BY 4.0 International license.

		Estimate	Est.Error	95% CI	
IFGorb	Sentence (Sent)	1.29*	0.18	0.93	1.64
	Backward Sent vs. Sent	-0.69*	0.12	-0.93	-0.45
	Nonsense Sent vs. Sent	-0.06	0.13	-0.31	0.21
	Word List vs. Sent	-0.87*	0.15	-1.15	-0.59
	Jab. Sent vs. Sent	-0.59*	0.12	-0.82	-0.35
	Nonword List vs. Sent	-1.09*	0.13	-1.35	-0.84
IFG	Sentence (Sent)	1.73*	0.16	1.42	2.07
	Backward Sent vs. Sent	-0.72*	0.13	-0.97	-0.46
	Nonsense Sent vs. Sent	0.10	0.13	-0.14	0.36
	Word List vs. Sent	-0.91*	0.13	-1.17	-0.65
	Jab. Sent vs. Sent	-0.89*	0.13	-1.14	-0.63
	Nonword List vs. Sent	-1.34*	0.14	-1.62	-1.07
MFG	Sentence (Sent)	2.31*	0.40	1.48	3.09
	Backward Sent vs. Sent	-0.27*	0.14	-0.55	-0.02
	Nonsense Sent vs. Sent	-0.05	0.14	-0.32	0.22
	Word List vs. Sent	-0.56*	0.14	-0.82	-0.29
	Jab. Sent vs. Sent	-0.63*	0.13	-0.90	-0.36
	Nonsense Sent vs. Sent	-0.05	0.14	-0.32	0.22
AntTemp	Sentence (Sent)	1.20*	0.10	1.02	1.40
	Backward Sent vs. Sent	-0.54*	0.07	-0.68	-0.41
	Nonsense Sent vs. Sent	-0.11	0.08	-0.26	0.04
	Word List vs. Sent	-0.73*	0.07	-0.86	-0.59
	Jab. Sent vs. Sent	-0.77*	0.08	-0.92	-0.62
	Nonword List vs. Sent	-1.04*	0.10	-1.22	-0.84
PostTemp	Sentence (Sent)	2.12*	0.16	1.80	2.42
	Backward Sent vs. Sent	-0.64*	0.10	-0.85	-0.44
	Nonsense Sent vs. Sent	-0.12	0.11	-0.33	0.10
	Word List vs. Sent	-0.90*	0.10	-1.10	-0.69
	Jab. Sent vs. Sent	-1.03*	0.11	-1.24	-0.81
	Nonword List vs. Sent	-1.37*	0.11	-1.59	-1.15

Table SI 5. The results of mixed effect linear regressions for the five language functional regions of interest (Results; Section 3.2). Condition was dummy-coded with *Sentence* as the reference level. IFGorb—orbital inferior frontal gyrus, MFG—middle frontal gyrus, AntTemp—anterior temporal lobe, PostTemp—posterior temporal lobe, Jab. — Jabberwocky.

bioRxiv preprint doi: https://doi.org/10.1101/2024.06.21.599332; this version posted June 22, 2024. The copyright holder for this preprint (which was not certified by peer review) is the author/funder, who has granted bioRxiv a license to display the preprint in perpetuity. It is made available under aCC-BY 4.0 International license.

	Estimate	Est. error	95% CI	
Sentence (Sent)	0.07	0.13	−0.19	0.31
Backward Sent vs. Sent	0.24*	0.09	0.41	1.01
Nonsense Sent vs. Sent	0.12	0.09	−0.04	0.29
Word List vs. Sent	0.22*	0.11	0.00	0.45
Jab. Sent vs. Sent	0.50*	0.09	0.33	0.67
Nonword List vs. Sent	0.36*	0.10	0.15	0.57

Table SI 6: Results of mixed-effects linear regression for fMRI responses within the Multiple Demand network. Stimulus type was dummy-coded with *Sentence* as the reference level. Sent — Sentence, Jab. — Jabberwocky. *Denotes significant difference.

bioRxiv preprint doi: https://doi.org/10.1101/2024.06.21.599332; this version posted June 22, 2024. The copyright holder for this preprint (which was not certified by peer review) is the author/funder, who has granted bioRxiv a license to display the preprint in perpetuity. It is made available under aCC-BY 4.0 International license.

Supplementary Methods

Behavioral incremental processing cost study

In this experiment, we measure the processing cost associated with the processing of grammatically well-formed sentences that convey plausible vs. unconventional meanings (i.e., the original sentences vs. the Nonsense versions of the sentences from the fMRI study).

Paradigm, and design and materials

We used the Maze self-paced reading paradigm (Forster et al., 2009; Boyce et al., 2020). In this paradigm, stimuli are revealed one word at a time, and at each time step, the correct (target) word is accompanied by a contextually inappropriate distractor word, and participants have to indicate the word that they believe is a more likely continuation via pressing one of two buttons (**Figure 4B**). Boyce et al. (2020) showed that reaction times (RTs) in this paradigm effectively capture incremental processing cost.

The experiment included two conditions: the *Sentence* and *Nonsense Sentence* conditions from the fMRI study (192 stimuli per condition; for details see Section 2.2.1). The 384 stimuli were distributed across 4 experimental lists (96 stimuli each, 48 per condition) such that each list contained only one condition of an item. In addition to the critical stimuli, each list included 4 practice items. To generate the distractor words for each time step of each stimulus, we used the automatic implementation of Boyce et al. (2020), where the distractors are real words that are not grammatically licensed by the preceding content.

Procedure

The experiment was implemented in the Maze module (Boyce et al., 2020) within the Ibex web-based psycholinguistic experiment software platform (https://github.com/addrummond/ibex).

The experiment began with detailed instructions. Following the instructions, participants completed 4 practice trials. Upon the completion of the practice trials, the critical experiment began. The 48 stimuli in each list were grouped into 6 blocks of 8 stimuli each, and participants were informed how many blocks remained after completing each block. To encourage participants to stay attentive throughout the experiment, a delay period of 2 s prevented participants' keypresses from registering whenever an error was made (for motivation, see https://vboyce.github.io/Maze/delay.html). The average completion time was ~10.5 min.

Participants

We recruited 80 participants through the Prolific web-based testing platform, restricting our task to participants with IP addresses in the United States. Participants were excluded from the analyses if their performance on the task was low (<80% accuracy; average accuracy was >90%). Data from 70 participants were included in the final analysis.
